# Advantages of Task-Specific Multi-Objective Optimisation in Evolutionary Robotics

**DOI:** 10.1371/journal.pone.0136406

**Published:** 2015-08-21

**Authors:** Vito Trianni, Manuel López-Ibáñez

**Affiliations:** 1 Institute of Cognitive Sciences and Technologies (ISTC), National Research Council (CNR), Rome, Italy; 2 IRIDIA, CoDE, Université Libre de Bruxelles (ULB), Brussels, Belgium; Peking University, CHINA

## Abstract

The application of multi-objective optimisation to evolutionary robotics is receiving increasing attention. A survey of the literature reveals the different possibilities it offers to improve the automatic design of efficient and adaptive robotic systems, and points to the successful demonstrations available for both task-specific and task-agnostic approaches (i.e., with or without reference to the specific design problem to be tackled). However, the advantages of multi-objective approaches over single-objective ones have not been clearly spelled out and experimentally demonstrated. This paper fills this gap for task-specific approaches: starting from well-known results in multi-objective optimisation, we discuss how to tackle commonly recognised problems in evolutionary robotics. In particular, we show that multi-objective optimisation (i) allows evolving a more varied set of behaviours by exploring multiple trade-offs of the objectives to optimise, (ii) supports the evolution of the desired behaviour through the introduction of objectives as proxies, (iii) avoids the premature convergence to local optima possibly introduced by multi-component fitness functions, and (iv) solves the bootstrap problem exploiting ancillary objectives to guide evolution in the early phases. We present an experimental demonstration of these benefits in three different case studies: maze navigation in a single robot domain, flocking in a swarm robotics context, and a strictly collaborative task in collective robotics.

## 1 Introduction

Artificial evolution is a powerful optimisation tool, and has been successfully applied to the synthesis of behaviours for autonomous robots, as demonstrated in the evolutionary robotics literature [1–4]. The advantages of the evolutionary robotics approach reside in the possibility of exploiting the sensorimotor coordination resulting from the interactions between the robot’s brain (i.e., the control software), its body (i.e., the embodiment including sensors and actuators) and the environment [5]. Through evolutionary approaches, the designer is exempted from a detailed modelling of the brain-body-environment interactions, and solutions can be obtained that match the specificities and statistical regularities of the problem at hand. However, a suitable engineering methodology to support the fundamental design choices in evolutionary robotics is currently missing. Indeed, most of the studies in evolutionary robotics strongly rely on the expertise of the designer, who assembles the evolutionary system following his personal intuition. Only few attempts have been made to propose an engineering methodology for the evolutionary design of robotic controllers [6, 7]. Among the several design choices required to devise an evolutionary robotics experiment, the fitness function is particularly important because it determines task-specific selective pressures to drive the evolutionary search (although task-agnostic approaches have been proposed as well [3, 8, 9]). However, the definition of a suitable fitness function is not always straightforward in evolutionary robotics [10, 11].

First of all, the features of the desired behaviour must be encoded in a measurable form, but often there is no definite and measurable way of expressing either the dynamical aspects of the robots’ behaviour or the desired outcome. Hence, it is common to find in the literature fitness functions composed of multiple *behavioural terms* that contribute to the one or the other feature (e.g., move fast, avoid obstacles, approach target) [11]. That is, the design problem in evolutionary robotics is intrinsically characterised by multiple objectives, but often tackled as a single-objective problem by means of an a priori aggregation (i.e., scalarization) of the various objectives. However, finding the correct trade-off between possibly conflicting terms is not easy. In this case, a multi-objective approach may provide a set of solutions that explore different trade-offs, so that a principled choice can be made a posteriori.

Secondly, the fitness function must support the *evolvability* of the system [12], that is, the possibility to progressively synthesise better solutions through random exploration and avoid premature convergence [3]. Even when a single-objective (fitness) function—or a scalarization of multiple objectives—is available for the desired behaviour, this function might be difficult to optimise by evolution, because it may present many local optima or suffer from the *bootstrap problem*, which is defined as the absence of selective pressures among randomly initialised individuals at the beginning of the evolutionary optimisation [11, 13, 14]. Hence, it may be preferable to adopt a multi-objective formulation and approximate the corresponding Pareto front (finding the actual Pareto front is typically infeasible in evolutionary robotics). In this case, the original objective function can be exploited for choosing *a posteriori* the best solution from the obtained Pareto set.

In the last two decades, evolutionary multi-objective approaches have shown their ability to explore multiple trade-offs in the objective space and to avoid premature convergence to poor solutions [15, 16]. As a result, the application of multi-objective optimisation (MOO) in evolutionary robotics is receiving increasing attention. However, evolutionary robotics goes beyond pure black-box optimisation, because there are multiple ways of introducing selective pressures other than the definition of the objectives to optimize [3]. As a consequence, MOO has been exploited in evolutionary robotics in several different ways to approach various challenges, and a survey is provided in Section 2. This survey highlights the lack of systematic studies that experimentally demonstrate the advantages of MOO, especially for what concerns the problems faced by designers in defining a suitable fitness function. In this paper, we fill this gap by casting well-known benefits of MOO approaches in terms of the problems they solve in evolutionary robotics, and by providing the first experimental demonstration about the advantages of MOO approaches in evolutionary robotics—as opposed to single-objective optimisation (SOO) approaches—in the context of the fitness definition problem. We name SOO approaches those algorithms that only try to find a single solution by either optimising a single fitness function or scalarizing multiple objectives a priori, i.e., before running the algorithm. Even though the study of the (a priori) scalarization of MOO problems is a subject studied in the MOO literature [17], the algorithms that actually tackle the scalarized problems, in the context of evolutionary robotics, do not differ substantially from those used to tackle genuinely SOO problems. By contrast, we name MOO approaches those algorithms that aim to approximate as best as possible the Pareto-optimal set. From this approximation set, the designer can choose, a posteriori, one behaviour as the final solution (see S1 Text for a brief introduction to MOO). This is a simplified view of MOO for the sake of comparison with the traditional SOO approaches used in evolutionary robotics. MOO in general includes a priori, a posteriori and interactive approaches [16–20].

Our experimental demonstration of the advantages of MOO over SOO is articulated in three case studies taken from the literature, which are conducted in simulation exploiting the ARGoS framework [21]. The first case study (Section 3) concerns the evolution of a navigation behaviour in a looping maze, following one of the pioneer studies in evolutionary robotics [22]. In this case, we show that MOO allows the evolution of a varied set of behaviours by exploring multiple trade-offs among the available behavioural terms. The second case study (Section 4) concerns another classic task: coordinated motion (flocking) with robots having only local perception of their neighbourhood. In this case, we show how MOO avoids the convergence to local optima induced by a multiple-components fitness function. Section 5 is dedicated to the third case study concerning a strictly collaborative task designed after a well-known experiment in collective robotics [23]. The problem requires multiple robots to simultaneously process objects scattered in the environment, and the performance of the system is measured as the number of objects processed. Collaboration is strictly required to perform this task. Consequently, a bootstrap problem may arise: a null fitness is assigned to behaviours that do not result in collaboration, which leads to the absence of selective pressures for randomly generated solutions. We show how the introduction of ancillary objectives bypasses the bootstrap problem and leads to the systematic evolution of satisfactory solutions. The paper is concluded with a discussion of the results and of the future research directions (see Section 6).

## 2 Modes of problem solving with MOO in evolutionary robotics

Multi-objective optimisation (MOO) can be exploited in evolutionary robotics in many different ways, according to the specific characteristics of the task and of the corresponding robotic behaviour to be synthesised (i.e., the design problem). In this paper, we refer mainly to the following three modes of problem solving with multiple objectives [18]: a) the design problem is a genuine multi-objective problem and should be treated as such; b) the design problem is tackled through a multi-objective approximation by proxies; or c) multi-objectivisation is applied to transform an actual single-objective problem into a multi-objective one. These modes of problem solving are not incompatible; on the contrary, they may appear conflated in practice. For example, a genuinely multi-objective design problem may contain one objective that is not easily defined as a fitness function, thus it requires the introduction of multiple proxy objectives, and another objective that is decomposed into several for the purpose of enabling its evolvability.

The above three modes of problem solving through MOO can be framed within the context of providing the right selective pressures to the evolutionary process. The different approaches to introduce/change selective pressures in evolutionary robotics—both SOO and MOO—are discussed by Doncieux and Mouret [3], who distinguish between approaches that change the requirements of the desired goal by changing the definition of the optimum solution (*goal refiners*), and approaches that help the evolutionary search process without actually changing the requirements of the task (*process helpers*). Within these two broad classes, they further distinguish between *task-specific* and *task-agnostic* approaches: the former require knowledge of the task and of the expected performance, while the latter provide methods applicable across different tasks. As we discuss in the following, MOO can be exploited both as a goal refiner and as a process helper. In both cases, MOO presents important advantages above SOO, yet these advantages have never been experimentally demonstrated through targeted case studies within the evolutionary robotics literature. Here, we discuss such advantages in the context of the above-mentioned three modes of problem solving with MOO, report examples from the literature, and indicate the missing evidence that our experimental studies provide.

### 2.1 Genuinely multi-objective problems

The desired behaviour of the robotic system may entail multiple requirements—different sub-goals—that may also be conflicting (e.g., they may represent the costs and benefits of applying a certain behavioural strategy). Therefore, a good trade-off must be found. Although the problem to be solved is a genuinely multi-objective optimisation problem, the classical approach in evolutionary robotics consists in the definition of a tailored fitness function that scalarizes, a priori, the different objectives (behavioural terms) to obtain a single-valued function [11], which can be optimised by means of well-known SOO algorithms. However, this scalarization—often a weighted sum—is somewhat arbitrary. The advantage of MOO is that it requires no such choice, and leaves the evolutionary process free to explore different trade-offs between the objectives, allowing the designer to choose a specific trade-off *a posteriori* on the basis of the analysis of the obtained solutions (see also Section 6 for a discussion about the selection of the best trade-off in relation to the evolutionary robotics domain). Given that the goal is to ultimately find the best trade-off between objectives, these MOO approaches can be considered goal-refiners, because they change the requirements of the task by introducing the Pareto optimality criterion and discarding, or not even defining, any a priori trade-off among objectives.

Most importantly, in evolutionary robotics different trade-offs may correspond to radically different behavioural strategies, which may not be attainable through single-objective evolution. Therefore, MOO leads to a large exploration of the possible solutions to a given robotics problem, and allows generating a wide set of behaviours, all optimising the trade-off between the given objectives. A simple MOO approach would be to optimise different scalarizations for a number of a priori defined weights, using SOO algorithms, and return the best solution found for each, in order to approximate the Pareto front. However, scalarizations based on weighted sums have well-known theoretical limitations [24]: (i) solutions that do not lay in the convex hull of the Pareto front are not optimal for any scalarization, and (ii) an even distribution of weights does not ensure an even distribution of solutions in the Pareto front. These limitations might not be crucial in practice for evolutionary robotics, since the Pareto front is often convex, the obtained solutions are just approximations to the optimal, and designers do not generally seek a perfectly even spread of solutions in the Pareto front but rather a sufficiently varied set of high-quality solutions. Nonetheless, we show later in the paper that, even for the typical scenarios of evolutionary robotics, such a simple MOO approach based on SOO algorithms is still inferior in practice to an MOO approach not relying on scalarizations.

Different research studies in the literature consider genuinely multi-objective problems. A first line of research exploits MOO to optimise at the same time the desired behaviour and some relevant task-specific feature of the controller, e.g., minimise the number of hidden units in a neural network [25, 26], or promote the efficient usage of state variables in a controller evolved trough genetic programming [27]. These studies demonstrate how MOO can be beneficial in identifying a good trade-off between the evolvability of the desired behaviour and the complexity of the controller. The latter should be high enough—to support the evolution of the desired behaviour—but not higher—to reduce the dimensions of the search space and ensure convergence. Teo and Abbass [25] present an extensive comparison between SOO and MOO approaches in which MOO approaches optimise both the behaviour and the controller structure, whereas SOO approaches fix the controller structure a priori and only evolve the behaviour. This comparison is, however, limited to one specific aspect of the advantages of MOO and does not address the problem of defining the correct fitness function to obtain a desired behaviour. Indeed, in the above studies the selective pressure to evolve the desired behaviour is provided by a single objective: if this objective is ill-defined or presents a low evolvability *per se*, no controller structure can help.

Another line of research exploits MOO for genuinely multi-objective tasks such as the evolution of virtual creatures displaying complex abilities, e.g., object grasping and legged locomotion [28, 29]. A genuinely multi-objective approach was also used to evolve complex morphologies of virtual creatures by introducing a cost for morphology complexity along with the reward for effective locomotion, with the goal of testing how different morphologies are evolved to match different environments [30]. Similarly, the parameters defining the morphology and kinematics of a flapping-wing robot have been optimised with a genuinely MOO approach [31]. The same flapping-wing robot has been evolved to show both target-following and altitude-control behaviours [32]. MOO was exploited to optimise the parameters of the controller of a four-wheeled mobile robot to perform aggressive maneuvers over slippery surfaces. A good trade-off was required between the two objectives representing the speed and accuracy in following the prescribed trajectory [33, 34].

A different line of research exploits MOO to evolve behaviours and robots with desired task-agnostic features. For instance, *transferability* of an evolved controller from simulation to reality is a much desired property, but it is difficult to obtain due to discrepancies between the simulation and the physical platform (often referred to as the *reality-gap problem* [3]). Thus, a possible approach consists in adding an objective that explicitly favours transferability [35], thus effectively expanding the design problem. A similar approach has been used for desired properties like morphological diversity [36], robustness through controller reactivity [37], resiliency to hardware failures [38], and generalisation to problem instances never encountered before [39]. The approaches used to obtain these properties are in general task-agnostic (e.g., search for novelty, behavioural diversity, transferability [8, 9, 35]), because the above properties do not depend on the given task but are generally desired across applications. The same approach has been used to evolve behavioural consistency and memory [40], although this particular approach may be considered task-specific because defining the additional objective requires knowledge about the scenarios for which the behaviour must be consistent.

These studies demonstrate that MOO can be successfully applied in an evolutionary robotics context, but they do not provide insights or experimental evidence about the advantages of MOO over SOO approaches. The only available comparison was performed by evolving a robot controller for two conflicting tasks: protecting another robot by remaining close to it and collecting objects scattered in the environment [41]. A genuinely MOO approach was compared against multiple scalarized problems obtained as the weighted average of the performance in the two sub-tasks, and solved using a SOO algorithm. The results did not reveal significant differences in the performance achieved by the two approaches, but noted the higher computational cost of running the SOO algorithm multiple times to produce different trade-offs between the two tasks [41]. In this paper, we provide an extensive comparison that better highlights the advantages of MOO over SOO across multiple tasks. In particular, we present a case study (see Section 3) where the multi-objectivization of an original SO fitness formulation does not provide additional evolvability, yet defining the design problem as a genuinely multi-objective problem disentangles the conflicting aspects of the fitness function. The multi-objective formulation leads to a larger diversity of solutions, which could not be obtained with the original SO formulation, and to a higher probability of discovering the desired behaviour.

In this paper, we limit our experimental studies to task-specific approaches, and we demonstrate how MOO improves the evolutionary search and, at the same time, obtains a larger behavioural diversity and a better exploration of the search space. We argue that these properties are delivered by MOO *per se*, and we provide an experimental demonstration by conducting a systematic comparison between single- and multi-objective evolution. We refer the interested reader to the studies mentioned above for what concerns approaches making use of task-agnostic techniques.

### 2.2 Multi-objective approximation by proxies

In evolutionary robotics, a suitable fitness function to optimise is often not available a priori, i.e., the task does not come with a detailed performance metric. Indeed, the real objective is the behaviour of the robotic system, and the fitness function represents just a means to obtain this behaviour. Evolutionary robotics represents a prototypical case in which “*it is the solution that has primacy, and the objectives are only a means of orienting the search in order to discover this solution*” [18]. In such conditions, the design problem can be faced by introducing multiple objectives that are proxies for the true “unavailable” objective. These proxies can be complementary, each covering some particular aspect of the behaviour to be obtained. “*Thus, it should be expected that the desired solution(s) will score relatively highly under all of the ‘proxy’ objectives, and an MOO approach may therefore be suitable*” [18].

Differently from genuinely multi-objective approaches, which assist in finding the (unknown) optimal aggregation among multiple objectives, multi-objective approximation by proxies should be counted among the task-specific *process helpers* [3]. The proxy objectives are actually intermediate goals or sub-goals that only indirectly optimise the overall goal, which is unavailable or insufficiently well defined. An MOO approach is particularly helpful in such conditions because the designer may not know a priori neither the relevance of each intermediate goal, nor how to aggregate them into a single fitness function. All the downsides of an a priori aggregation mentioned above become even more critical here, because no aggregation of the multiple objectives measures the desired behaviour. In practice, determining if an evolutionary robotics study reports a genuinely multi-objective problem or an approximation by proxies may be somehow arbitrary, because it is sometimes unclear whether an objective is a pre-existent requirement or just a proxy.

One of the first applications of MOO in evolutionary robotics concerned the incremental evolution of controllers for an unmanned aerial vehicle (UAV) through multi-objective genetic programming [42]. In this case, the overall goal was too complex to be described by a particular objective (or even to be evolved using a single evolutionary stage), since the UAV was required to locate, reach and track a radar source, hence, multiple ancillary objectives were defined for evolving the behaviour in several stages. A clear example of task-specific proxies introduced to evolve a desired behaviour is given by Moshaiov and Ashram [43] when tackling a single-robot navigation problem (a problem inspired by the same pioneer study we refer later in this paper). In their case, an additional objective with respect to the original fitness formulation was introduced to reward the passage through pre-defined waypoints, in order to favour a looping behaviour. A similar approach was used when comparing multi-objective evolutionary strategies and genetic algorithms, in which a robot was required to display both collision-free navigation and object retrieval abilities [44]. Another relevant study concerns the evolution of a walking controller for a humanoid robot [45, 46]. In that study, several proxies are introduced that reward specific characteristics of the desired gait, still the real objective is efficient locomotion. Task-specific proxies have also been used to evolve efficient navigation employing an artificial potential field [47], in which the proxies rewarded different aspects of the navigation ability, such as goal approaching or obstacle avoidance.

In this paper, we discuss a flocking case study in Section 4 that demonstrates how multi-objective approximation by proxies is a viable method to devise the selective pressures required to obtain a desired behaviour. We also show that it is superior to the a priori scalarization of the same objectives.

### 2.3 Multi-objectivisation

Even when a single-objective function is available for measuring the performance in the given robotics problem, it may not be suitable for evolutionary optimisation because it may not provide evolvability to the system. Generally speaking, the evolvability issue is related to the search landscape of the optimisation problem, which can be either very rugged, presenting many local optima, or on the contrary largely flat, offering no gradient for the evolutionary search. The latter case gives rise to the *bootstrap problem*, which is the absence of selective pressures among randomly initialised individuals at the beginning of the evolutionary optimisation [11, 13, 14].

Multi-objectivisation is a technique that allows transforming a difficult SOO problem into a more tractable MOO problem [18] by either decomposing the SOO problem into multiple objectives in order to disentangle the concurrent aspects giving rise to local optima, or by introducing ancillary objectives to kick-start or guide the evolutionary search. Both the decomposition and the ancillary objectives are process helpers, as they are just relevant for guidance, and the final solution(s) may largely ignore them, focusing on the original objective alone. The key distinction between multi-objectivisation and multi-objective approximation by proxies is the existence of an original objective that measures the final design. In the latter case, such objective does not exist or it is not sufficiently well-defined. In practice, this distinction depends on what the goal of the designer is.

Multi-objectivisation has been exploited by adding both task-specific and task-agnostic objectives used for guidance. In [48], an ancillary task-specific objective was added to provide hints on how to solve a complex task. This objective rewarded the usage of a knowledge base of behaviour segments automatically generated on a simpler version of the same task. In recent interactive evolutionary experiments [49, 50], the age of the genotype was exploited to promote the preservation of novel solutions and give them a chance to be evaluated by the interactive users. In these experiments, the feedback from the user was not considered as an additional helper objective and the experiments used a simple scalarization. Other recent studies [9, 51, 52] exploited MOO in conjunction with techniques to maximise behavioural diversity among the evolved solutions, to promote a better exploration of the search space and tackle the bootstrap problem. In these studies, one objective was dedicated to the fitness of the goal-task, while a second objective corresponded to a diversity measure. An extensive comparison of SOO and MOO approaches for encouraging behavioural diversity, varying also the controller structures and the diversity measures, showed that MOO performs best in such cases [9].

Multi-objectivisation has been exploited to tackle the bootstrap problem in evolving controllers for complex incremental tasks, that is, tasks that require the completion of multiple sub-tasks to achieve the main goal [13]. In similar conditions, it is possible to define a MOO problem in which the different objectives correspond to the achievement of the individual sub-tasks. The only limiting factor of this otherwise well grounded work resides in the experimental scenario, since the existence of clearly defined sub-tasks that can be characterised as conflicting objectives explicitly favours MOO over SOO approaches. In this paper, we provide further evidence on the relevance of MOO to tackle the bootstrap problem in a task that intrinsically leads to low evolvability, but that does not prevent SOO to find a solution (see Section 5). In this way, we extend the demonstration of the benefit of MOO over SOO as a general approach for evolutionary design methods.

## 3 Case Study: Navigation in a Maze

The first case study corresponds to the evolutionary design of a navigation behaviour for a single robot to explore a looping maze. We choose this case study as it represents a necessary benchmark to test the suitability of a design methodology, it is simple enough to be fully analysed and understood, and it has been widely studied in the past. We start from a classic study in evolutionary robotics [22], which proposed a simple setup to perform the evolutionary optimisation of a neural network controller directly on the real robotic hardware. The physical setup puts several constraints on the information available to compute the performance, as absolute positions are not available to the robot. The fitness function has been therefore defined exploiting solely the information accessible to the robot, that is, the infra-red (IR) proximity sensors to account for the distance from obstacles, and the wheels’ encoders to account for the robot motion speed. In our work, despite experiments are performed in simulation, we adopt the same rationale as if the fitness was computed on the physical robot (i.e., the *marXbot* [53]) to keep the setup coherent with the original study [22].

**Fig 1:**
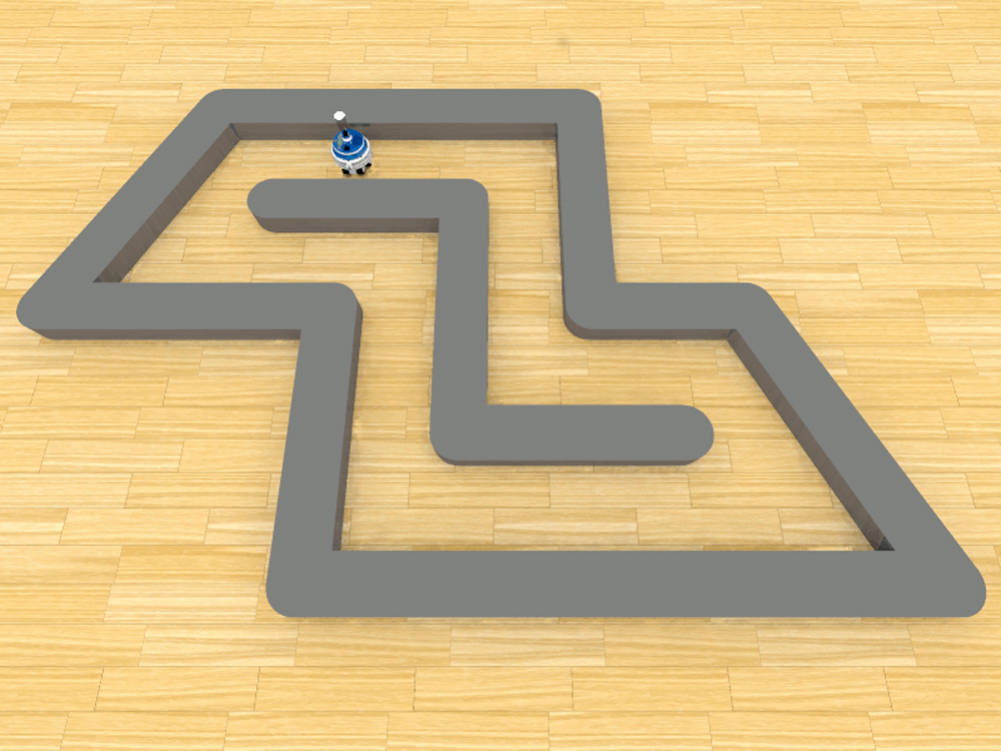
Setup of the navigation experiment. The looping maze is characterised by both wide and narrow corners, which challenge the navigation abilities of the robot. The robot is initialised randomly within the looping maze and with a random initial orientation. Experiments are performed in simulation exploiting the ARGoS framework [21].

The desired behaviour for the robot is fast motion and obstacle avoidance in a closed circuit, and represents an instance of a genuinely multi-objective problem. In this work, the circuit has been designed to replicate the main features of the experimental arena used in the original study [22], which is characterised by left and right turns, as well as wide and narrow corners (see Fig 1). The desired behaviour would efficiently negotiate corners and narrow passages, and would result in a smooth navigation to cover the whole circuit. With this goal in mind, we decided to evolve a simple reactive neural network controller. Therefore, no path planning or trajectory tracking has been used, and the navigation strategy directly derives from the evolved neural controller. The neural controller and the experimental setup is detailed in S1 Text. We use the original formulation for the fitness function, and contrast it with a two-objective formulation derived from the original one (Section 3.1). Additionally, we compare the MOO approach and a SOO approach based on a scalarization through weighted sum of the two objectives. The methodology for comparing single and multi-objective approaches is presented in Section 3.2, and the obtained results are discussed in Section 3.3.

### 3.1 Fitness function and multi-objective formulation

The original fitness function is designed to reward fast motion and obstacle avoidance on the basis of the information available to the robot. Therefore, at each control step *t*, the current angular speed of the wheels and the readings from the IR proximity sensors are collected, and a single-objective performance measure is computed as follows:

$$N_{T}=\frac{1}{T}\sum_{t}\Omega \left(t\right)\cdot \Phi \left(t\right)$$ (1)

where *T* is the total number of control steps. Ω(*t*) is the reward for moving straight and fast at time *t*, and is computed on the basis of the current angular speed of the robot wheels:

$$\Omega \left(t\right)=\frac{|\omega_{l}\left(t\right)|+|\omega_{r}\left(t\right)|}{2\omega_{m}}\cdot (1-\sqrt{\frac{|\omega_{l}\left(t\right)-\omega_{r}\left(t\right)|}{2\omega_{m}}}),$$ (2)

where *ω*_*l*_ and *ω*_*r*_ are the left- and right-wheel angular speeds, and *ω*_*m*_ is the maximum possible speed of the differential-drive motion controller. Here, the first factor rewards fast motion because it is maximised by high absolute values of the angular speed. The second factor rewards straight motion as is maximised by small differences between the left and right angular speeds. Φ(*t*) is the reward for being away from any obstacle at time *t*:

$$\Phi \left(t\right)=1-\max_{i}\frac{\phi_{i}\left(t\right)}{\phi_{m}},$$ (3)

where *ϕ*_*i*_(*t*) is the activation of the *i* ^*th*^ IR proximity sensor, and *ϕ*_*m*_ is the maximum possible value. The closer the obstacle, the higher the sensor activation, the smaller the reward. 𝓝_𝓣_ is a scalarization that averages over time the product of these two components, therefore requiring that both components are non-null for a large amount of time. The interesting aspect of this function is that both its components are low at the same time while the robot is negotiating an obstacle. Therefore, 𝓝_𝓣_ is maximised by controllers that ensure a fast reaction to the perception of an obstacle that leads the robot away from it.

Starting from the above formulation, we define two objectives by decomposing the function 𝓝_𝓣_ as follows:

$$\begin{array}{ccc} N_{\Omega } & = & \frac{1}{T}\sum_{t}\Omega \left(t\right), \end{array}$$ (4)

$$\begin{array}{ccc} N_{\Phi } & = & \frac{1}{T}\sum_{t}\Phi \left(t\right). \end{array}$$ (5)

The objective 𝓝_Ω_ encodes information about the wheels speed over time, and corresponds to the evaluation of the robot’s ability to move straight and fast. On the other hand, 𝓝_Φ_ encodes information about the proximity to obstacles and walls, and is maximised by behaviours that keep the robot away from them. The two objectives introduce an interesting trade-off, that was not considered in the original formulation. Indeed, there may be behaviours that move less but stay maximally away from obstacles, or fast-moving robots that however keep the walls closer. As we will see below, the MOO approach will explore these possible trade-offs producing several qualitatively different behaviours.

Finally, we introduce a single-objective formulation that scalarizes the two objectives introduced above by means of a weighted sum, as follows:

$$N_{\gamma }=\gamma N_{\Omega }+\left(1-\gamma \right)N_{\Phi }$$ (6)

with varying *γ* ∈ [0, 1]. The rationale is that different trade-offs between the two objectives can be explored by varying *γ*, but we can still use the SOO approach to optimize Eq (6) for each value of *γ*. Although such an approach has well-known theoretical limitations [24], it could be in practice more efficient in finding alternative solutions to the navigation problem than the purely MOO approach.

### 3.2 SOO vs. MOO

To compare the SOO and MOO approaches, we run several repetitions of the same evolutionary algorithm, changing only the way in which potential solutions are evaluated and selected for reproduction. In the SOO case, solutions are selected according to the fitness function. In the MOO case, solutions are selected according to the “hypervolume measure”, which represents the “*size of the dominated space*” [54]. This type of selection is used by state-of-the-art multi-objective evolutionary algorithms [55, 56], but here we just replace the selection step of a standard SOO algorithm. A detailed description of the evolutionary algorithm is available in S1 Text. For each experimental condition, we run twenty evolutionary runs for a fixed number of generations, and we consider the solutions of the last generation for further analysis. All solutions are re-evaluated to obtain an unbiased assessment of their performance using the available performance metrics, i.e., the (scalarized) fitness and the individual objective measures. The comparison between the SOO and MOO approaches is carried out on these measures by looking at the probability that a specific approach dominates the other one. All the details of the comparative approach we employ are available in S1 Text.

### 3.3 Results

#### 3.3.1 Performance analysis

**Fig 2:**
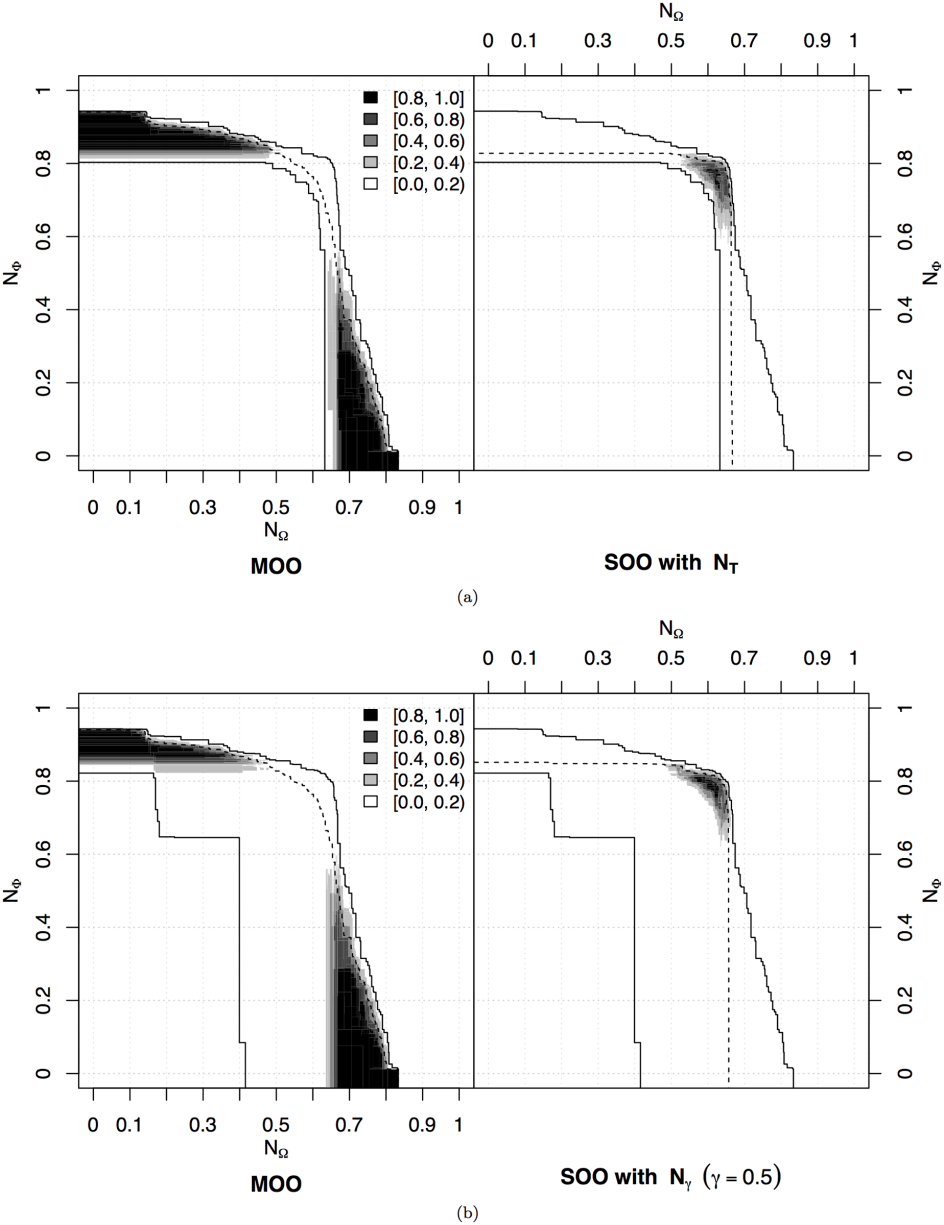
Navigation experiment. Differences between the MOO and the SOO approach with (a) the traditional fitness function 𝓝_𝓣_ and (b) the fitness 𝓝_*γ*_, with *γ* = 0.5. The comparison is carried out by means of *empirical attainment functions* (EAFs) [57–59], which give the (estimated) probability that a single run of an optimiser *attains* (dominates or equals) a specific point in the objective space (for details, see S1 Text). The difference between EAFs is reported in the figure, where grey-levels represent the magnitude of the difference: darker colours indicate larger differences. For example, a black point on the left indicates that the difference between the EAF of the left optimiser minus the EAF of the right optimiser is at least 0.8, that is, the left optimiser has attained that point in at least 80% more runs than the right one. The solid lines shown in the plot delimit the points attained, respectively, in at least one run (grand-best attainment surface) and in every run (grand-worst attainment surface) by any of the two optimisers. Any difference between the optimisers is located between these two lines. The dashed lines, which are different on each side, delimit the points attained in 50% of the runs (median attainment surface) of the optimiser shown in that side.

Following the methodology described above, we compare the SOO and MOO approaches by looking at the solutions in the objective space given by 𝓝_Ω_ and 𝓝_Φ_. The comparison between the MOO approach and the SOO approach using the traditional fitness formulation 𝓝_𝓣_ is given in Fig 2a. Here, we plot the differential ability of the two approaches in attaining portions of the objective space. It is possible to observe that the solutions found by the SOO approach prevail only in the region in which both speed and distance from obstacles are maximised (see the dark areas on the right part of Fig 2a). This region is also attained by the MOO approach, but less frequently. Indeed, the MOO approach explores widely the objective space, and proves best in all the other regions in which either speed or distance from obstacles are maximised. These regions are never attained by the single-objective evolution. By looking at these results, we can conclude that MOO does not provide a competitive advantage over SOO if we are only interested in the original single-objective performance. A very similar discussion can be done for the comparison with the weighted sum 𝓝_*γ*_, with *γ* = 0.5. Also in this case, the single-objective approach finds and optimises only solutions that maximise at the same time the two objectives, being deficient in the other parts of the objective space (Fig 2b).

**Fig 3:**
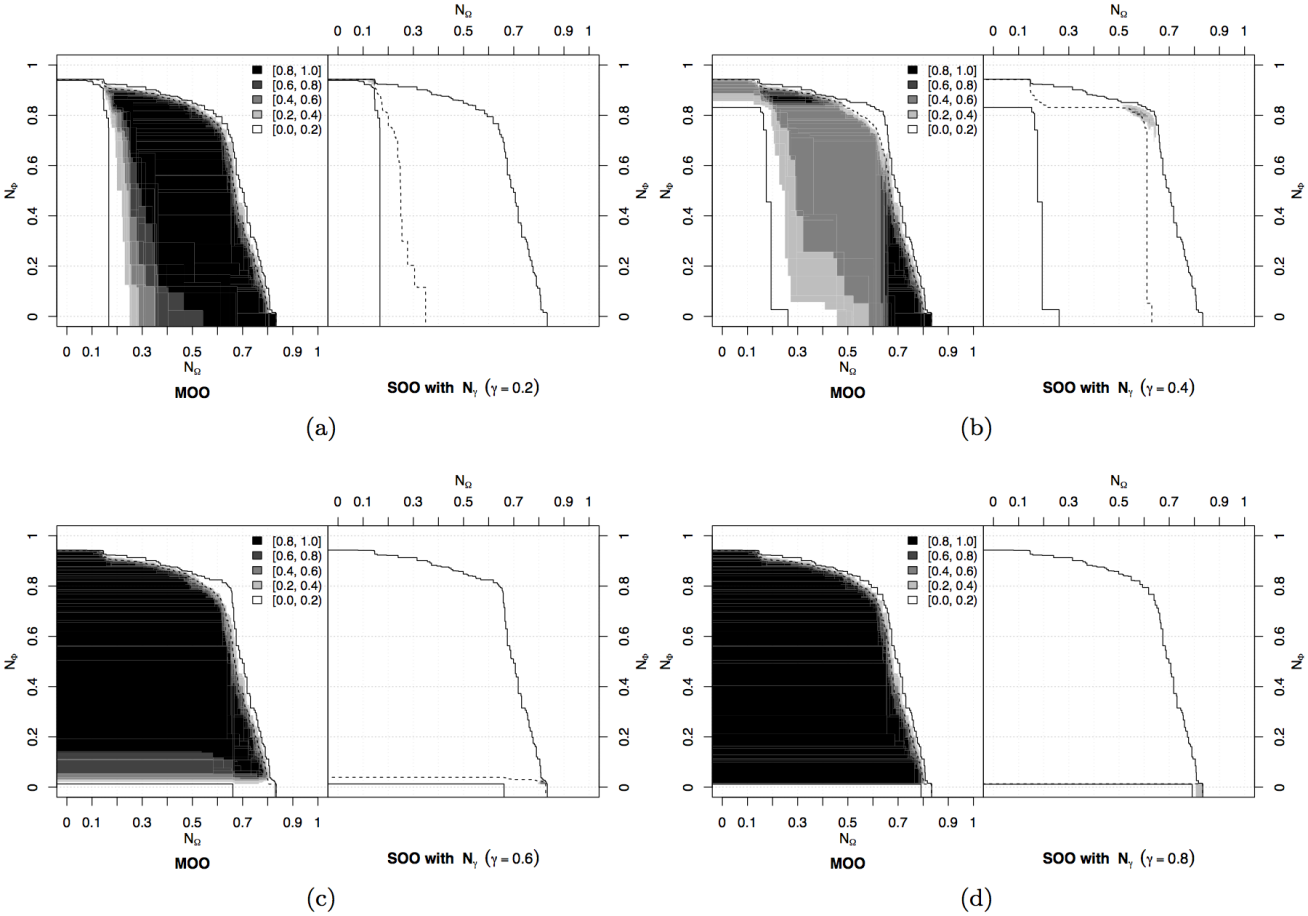
Navigation experiment. Differences between the EAFs of the multi-objective approach and the single objective approach with weighted fitness 𝓝_*γ*_ and varying weight *γ* between components (see caption of Fig 2 for an explanation of the EAFs difference).

The situation is different if we vary the weighted sum of the two objectives. We tested different weights by systematically varying *γ*, as shown in Fig 3. In this case, the MOO approach has a large advantage on most parts of the objective space, generally dominating the weighted sum solutions. For *γ* = 0.2, mainly obstacle avoidance is rewarded, and single-objective evolution remains trapped in a local optimum in which only 𝓝_Φ_ is maximised (Fig 3a). Conversely, for *γ* = 0.6 and *γ* = 0.8, SOO mainly finds the other local optimum in which only 𝓝_Ω_ is maximised (Fig 3c and 3d). In all these cases, the SOO approach does not produce good solutions for the navigation problem. The weight *γ* = 0.4 provides better results, with the SOO approach showing a slight advantage in attaining areas of the objective space close to the central part of the Pareto front (Fig 3b), but this looks less efficient than the case for *γ* = 0.5 shown in Fig 2b. These results demonstrate that the choice of the right compromise between different objectives is very difficult, and even relatively small variations may lead to radically different results. This aspect is discussed further in the case study presented in Section 4.

#### 3.3.2 Behaviour diversity

As expected, individual single-objective runs do not explore different trade-offs. With 𝓝_𝓣_, the selective pressure is focused on the region of the objective space where 𝓝_Ω_ ≈ 𝓝_Φ_. In these conditions, the evolved solutions produce behaviours that present at the same time high values of Ω(*t*) and Φ(*t*). This happens when a robot moves as fast as possible and as far as possible from obstacles. When a corner must be negotiated, the evolved strategy makes the robot quickly rotate on the spot. In particular, we observed that the avoidance action is performed by turning always in the same direction, notwithstanding the maze requiring a left or a right turn. This leads to a fast and efficient collision-avoidance action, because the robot does not need to determine whether the turn is on the left or on the right. However, despite optimal with respect to the single-objective function, this strategy trades the avoidance speed with the exploration abilities in the maze. In fact, the robot remains trapped in some parts of the circuit because it is unable to correctly negotiate both left and right turns. Evolving the neural network controllers with 𝓝_𝓣_ produces only behaviours of this kind, which we will refer to as *quick-avoidance* behaviours. An example of this behavioural strategy is provided by S1 Video.

**Fig 4:**
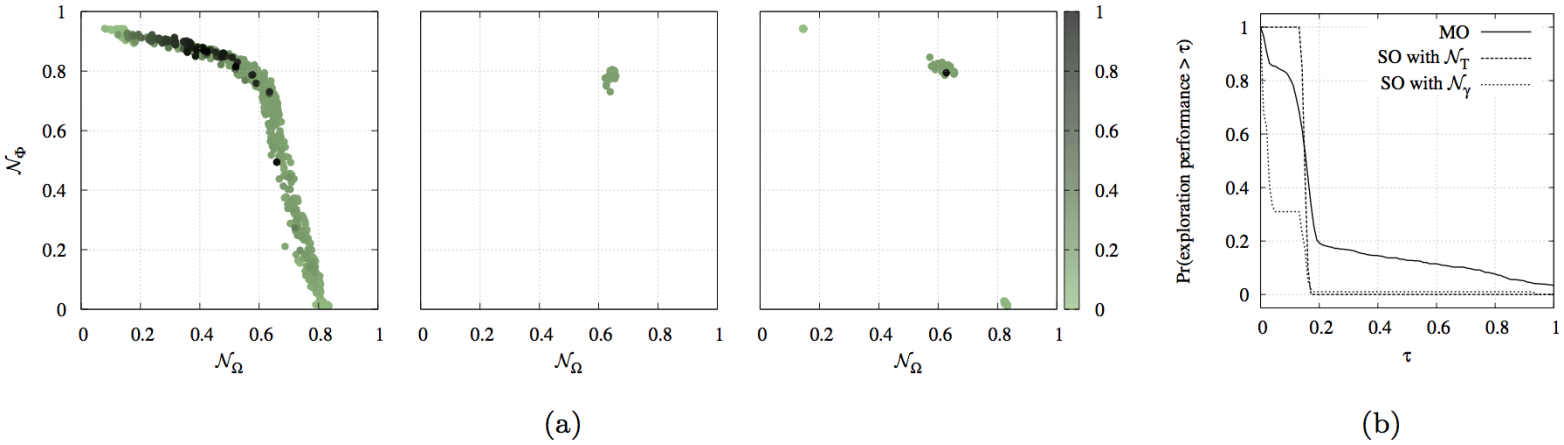
Navigation experiment. (a) The exploration ability of the evolved controllers with the MOO and the SOO approaches. Each dot represents a solution in the objective space, and the darkness of the circle corresponds to the exploration performance, measured as the fraction of the maze that was effectively covered by the robot. (Left) Solutions obtained with MOO. (Centre) Solutions obtained with SOO using 𝓝_𝓣_. (Right) Solutions obtained with SOO using 𝓝_*γ*_ for every *γ* ∈ {0.2,0.4,0.5,0.6,0.8}. (b) Empirical tail distribution of exploration performance, that is, probability that the exploration performance is larger than a given performance threshold *τ*.

Multiple scalarizations with 𝓝_*γ*_ provide an approximated Pareto set, but are not able to cover the whole front [24]. On the contrary, the MOO approach explores the objective space widely and provides a better approximation of the Pareto set, therefore producing behaviours of different kind that optimise different trade-offs of 𝓝_Ω_ and 𝓝_Φ_ (see also Fig 4a). By observing the behaviour corresponding to the different solutions found, we can identify mainly four categories:

- the solutions that maximise 𝓝_Ω_ only, which correspond to controllers that make the robot move fast, but do not present any obstacle avoidance ability;
- the solutions that maximise 𝓝_Φ_ only, which correspond to controllers that keep the robot away from obstacles, but do not present any navigation ability;
- the solutions that mainly maximise 𝓝_Ω_, possibly at the cost of a lower 𝓝_Φ_, which correspond to controllers producing the quick-avoidance behaviour described above;
- the solutions that mainly maximise 𝓝_Φ_ at the cost of a lower 𝓝_Ω_, which correspond to robots that move following the closest wall, either on the left or on the right. This *wall-following* behaviour leads to a very good exploration of the maze, which can be performed at the cost of a reduced speed in order to keep a constant distance from the wall (see also S1 Video).

For the engineering of good navigation in a maze, the MOO approach has the advantage of providing a varied set of possible solutions in each evolutionary run. Some of the evolved behaviours are not useful for navigation purposes, such as those belonging to the categories (i) and (ii) described above. However, the *wall-following* behaviour clearly outperforms the *quick-avoidance* behaviour in terms of exploration of the maze, and represents the natural choice that would be made *a posteriori* by a designer interested in good maze-navigation strategies. This gives MOO an advantage over SOO approaches that can be quantified. To this purpose, we compute an exploration performance value that measures the exploration abilities of the best evolved solutions by looking at the percentage of the maze that is visited by the robot in a sufficient amount of time to cover the full length of the circuit. Fig 4a shows the exploration performance for all the solutions obtained with MOO and SOO. Note that we consider all the solution obtained with 𝓝_*γ*_ as a unique experimental condition, in the attempt to approximate the Pareto set by systematically varying *γ*. The best controllers have been chosen in the MOO case from the approximated Pareto set of the different evolutionary runs, while in the SOO case they where selected as the solutions with the highest average performance. This corresponds to the choice one would make *a priori* when selecting or discarding solutions from the last population, as described in S1 Text. Fig 4a shows that all the best solutions of the SOO approach with 𝓝_𝓣_ present poor exploration, as they all belong to the quick-avoidance category. Similarly for the case of SOO with 𝓝_*γ*_, even though a few solutions with good exploration can be observed. Instead, in the MOO case, a large fraction of solutions provide a good exploration, suggesting that the wall-avoidance behaviour was often discovered.

Given that the MOO approach produces much more solutions than the SOO approach, we can compare their efficiency by looking at the probability of obtaining a solution presenting a good exploration behaviour. We estimate this probability by computing the fraction of evolved solutions that present an exploration performance higher than a threshold *τ*, at varying *τ*. We plot this probability as an empirical tail distribution (i.e., the complementary of the cumulative distribution function) in Fig 4b. We note that 𝓝_𝓣_ produces only solutions that visit less than 20% of the maze, which corresponds to poor exploration abilities. For larger *τ*, the probability of obtaining good navigation behaviours is higher for the MOO approach than for 𝓝_*γ*_, which only occasionally produces the wall-following behaviour. Thus, we conclude that MOO delivers better results for what concerns the diversity of behaviours evolved, even when compared against a scalarization approach exploring various values of *γ*. In this particular case, better exploration also corresponds to the discovery of desired solutions that could not be easily obtained with the SOO approach.

## 4 Case Study: Flocking

Flocking is a standard behaviour extensively studied in swarm intelligence and swarm robotics. It requires that independent, autonomous agents in a group move coordinately on the basis of local information only. By observing the dynamics of bird flocks and fish schools, several theoretical models have been developed to describe the individual rules underlying coordinated motion [60, 61]. Generally speaking, three simple rules are sufficient: (i) collision avoidance, (ii) flock centering and (iii) velocity matching. The first two rules provide the means to achieve and maintain *cohesion* in the group, because agents are attracted to each other while maintaining a safety distance. Velocity matching instead makes an agent orient itself in the average direction of the neighbours, eventually leading to the alignment of all individuals, which is necessary for efficient group *motion*. The execution of these rules is possible on the basis of local information only—distance, bearing and heading of close neighbours—and is sufficient for the establishment and maintenance of coordinated motion.

**Fig 5:**
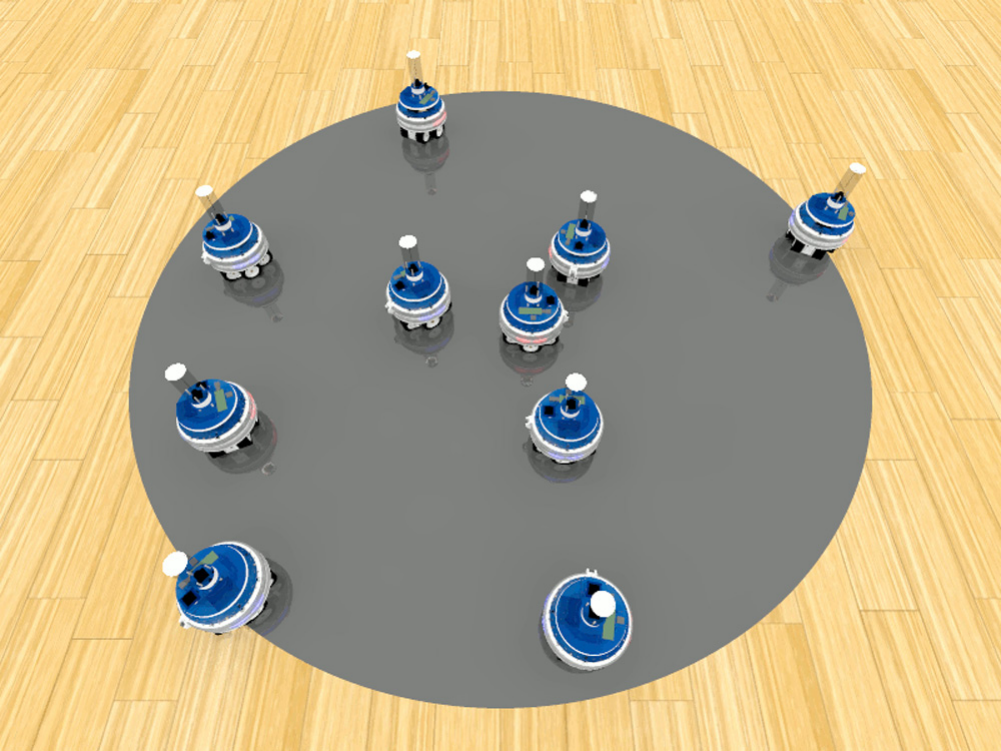
Setup of the flocking experiment. Ten robots are initialised randomly within a circle of 1 m radius (see grey circle) with random orientation. Each robot displays a coloured left-right pattern with red and blue LEDs to provide information about its heading direction (see S1 Text for more details). Experiments are performed in simulation [21].

The evolution of a coordinated motion behaviour represents a prototypical case in which the desired behaviour of the group is well understood, but a fitness function to evolve it is not available (see for instance [62, 63] for two alternative approaches). The description of the flocking behaviour naturally yields to a multi-objective formulation: the group must move as far as possible (maximise motion) while keeping coherence (maximise cohesion). These can be considered as “proxies” to guide the evolutionary search toward desired solutions. We therefore decided to contrast a MOO approach using these two metrics with the SOO approach derived from scalarization through weighted average of the two proxies (see Section 4.1 for details). Ten robots are initialised within a circle of 1 m radius with random position and orientation (see Fig 5). The robots use their coloured LEDs to display a left-right pattern that provides some information on their heading, which can be used for aligning the motion direction. A complete description of the experimental setup for this case study is available as supplementary material in S1 Text, while the obtained results are discussed in Section 4.2.

### 4.1 Motion and cohesion objectives and fitness function

In order to reward motion and cohesion in a group, the simplest way is to rely on the absolute positions of the robots, which are available in our simulation environment. At the beginning of a trial, robots are placed within a circle of 1 m radius, choosing their position and orientation at random. To reward group motion, it is sufficient to look at the displacement of the centre of mass of the group during a fixed-duration trial:

$$F_{m}=\frac{∥\hat{\text{X}}\left(T\right)-\hat{\text{X}}\left(0\right)∥}{D_{m}\left(T\right)},$$ (7)

where $\hat{\text{X}}\left(t\right)$ is the position of the centre of mass at time *t*, *D*_*m*_(*t*) is the maximum distance a single robot can travel in *t* seconds, and *T* is the length of a trial. Cohesion instead is maximised when the average distance of the robots from the centre of mass of the group is minimised:

$$F_{c}=\max(0,1-\frac{1}{N}\sum_{i}\frac{∥{\text{X}}_{i}\left(T\right)-\hat{\text{X}}\left(T\right)∥}{d_{m}}),$$ (8)

where **X**_*i*_(*t*) is the position of robot *i* at time *t*, *N* is the total number of robots, and *d*_*m*_ is the maximum tolerated distance. Cohesion is computed at the end of the trial, assuming that the group must remain aggregated for the whole duration of the trial (and possibly beyond it).

Also in this case, we can obtain a single-objective formulation from the two above objectives through a scalarization:

$$F_{\gamma }=\gamma F_{m}+\left(1-\gamma \right)F_{c},$$ (9)

with *γ* ∈ [0, 1]. To properly select the weight *γ*, we would need to know *a priori* the relative importance of the two components of the fitness. Since this relative importance is generally unknown, the standard approach is therefore to systematically vary *γ*. Consistently with the previous case study, we provide here the results for *γ* ∈ {0.2,0.4,0.5,0.6,0.8}.

### 4.2 Results

**Fig 6:**
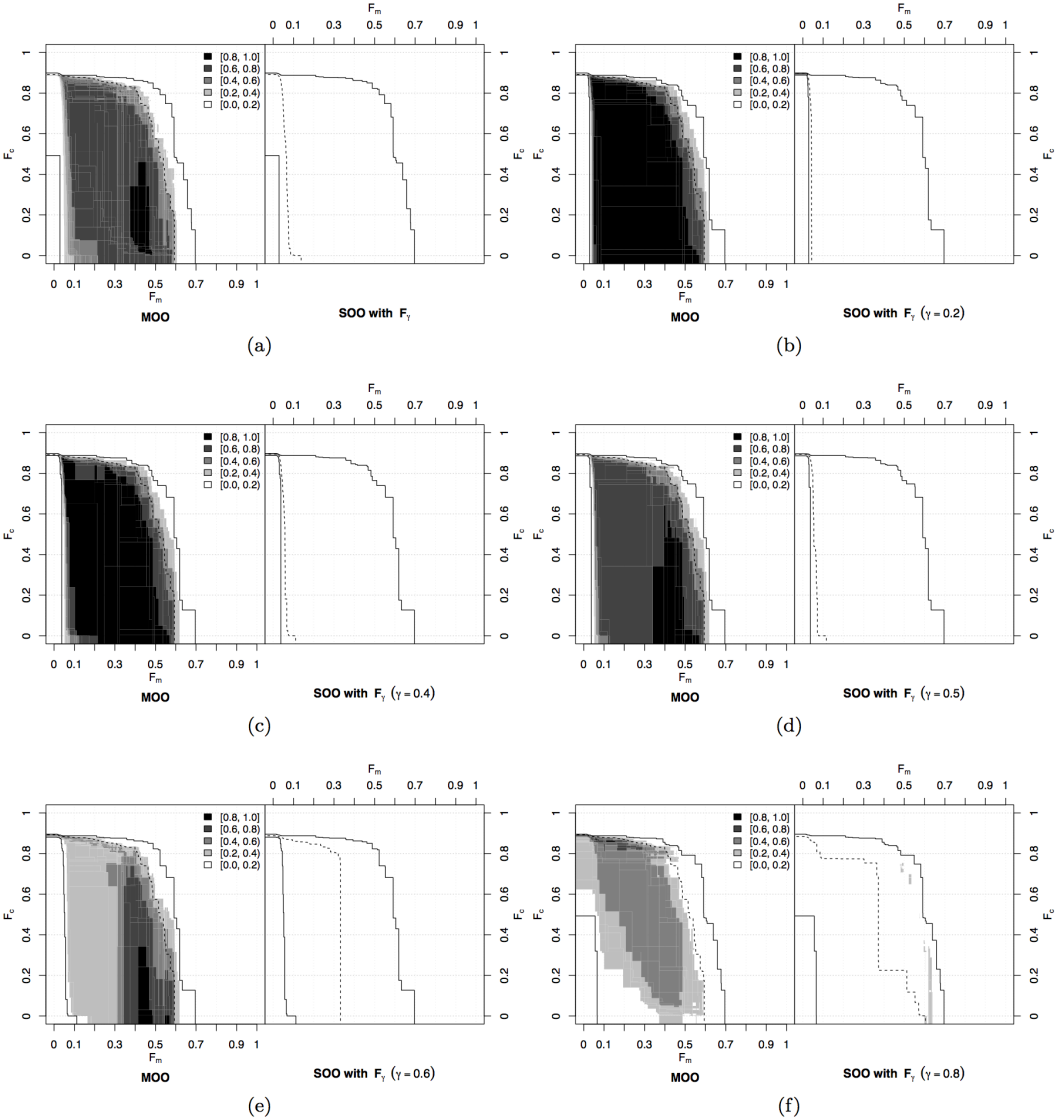
Flocking experiment. Differences between the EAFs of the MOO approach and the SOO approach with weighted fitness 𝓕_*γ*_. (a) Comparison with the combined results of all scalarizations. (b-f) Comparison for specific values of the weight *γ* between components (see caption of Fig 2 for an explanation of the EAFs difference).

We again followed the methodology described in Section 3.2 and detailed in S1 Text. The results of the comparison between the different approaches is presented in Fig 6. Generally speaking, the MOO approach outperforms the SOO approach with 𝓕_*γ*_, presenting a higher probability of attaining all regions of the objective space (Fig 6a). If we look at the comparison with specific values of *γ* as shown in Fig 6b to 6f, only for *γ* ≥ 0.6 the differences fall below 60% in favour of the MOO approach for most of the objective space (see Fig 6e). By contrast, the choice of *γ* = 0.5 (the natural choice one would make without any a priori knowledge) results in poor performance, with most of the objective space attained by the MOO approach with at least 60% more probability than the SOO approach. The reason is that low values of *γ* bias the search towards solutions presenting high cohesion and no motion. These solutions behave as local optima from which it is difficult to escape, even when more importance is given to the motion objective 𝓕_*m*_ with higher values of *γ*. Indeed, solutions providing only cohesion and no motion are obtained also with *γ* = 0.8.

**Fig 7:**
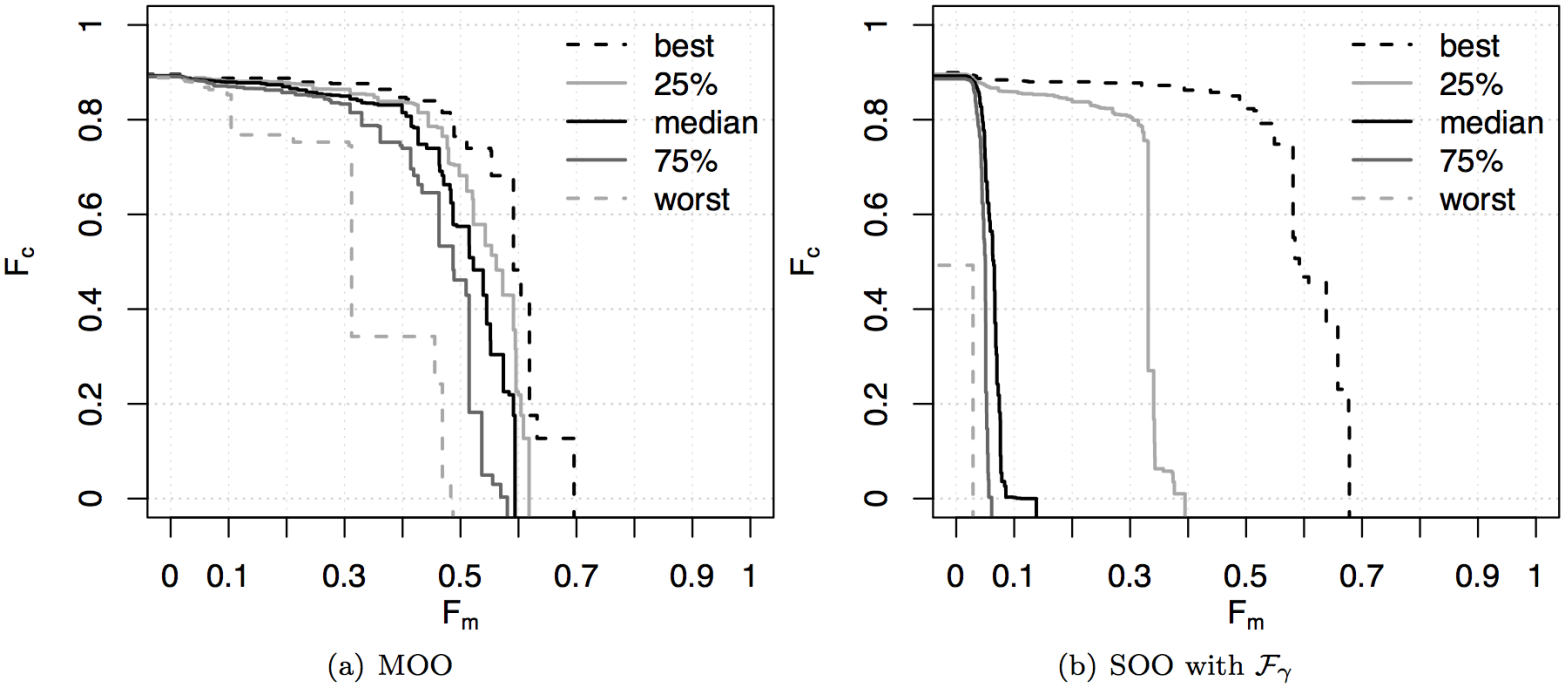
Flocking experiment. Attainment surfaces of (a) the MOO approach and (b) the SOO approach for all values of *γ*. The *k*%-attainment surfaces shown in the figure allow visualising the EAF over the objective space [59]. As explained earlier, the EAF indicates the (estimated) probability of dominating a point in the objective space in a single run of an algorithm. A *k*%-attainment surface denotes the Pareto front of the points that have been attained by (dominated by or equal to) at least *k*% of the runs, that is, the points that have a value of at least *k*/100 of the EAF. Hence, the worst attainment surface indicates the Pareto front of the points that have been attained in 100% of the runs. Conversely, the best attainment surface indicates the Pareto front of the points that have been attained by at least one run. More details on the EAFs are available as supplementary material in S1 Text.

The performance argument alone gives already a point in favour of the MOO approach, as it proves capable of attaining more frequently all areas of the objective space. However, not all possible trade-offs correspond to good coordinated motion behaviours. From an engineering perspective, those solutions providing low motion or low cohesion should not be retained: the former correspond to packed groups that do not move, the latter correspond to groups that split by moving in multiple directions. It is therefore useful to look at the probability of producing acceptable solutions by using the SOO and MOO approaches. To this purpose, we examine the attainment surfaces of both approaches as they give an absolute indication about the algorithm ability to produce solutions with a given trade-off between the objectives (Fig 7). Here, we compare the MOO approach with the SOO approach aggregating all values of *γ*. While the best attainment surfaces are comparable, the probability of producing solutions that maximise both objectives at the same time is lower for the single-objective approach. With the MOO approach, even the worst attainment surface contains solutions that feature reasonably good cohesion and motion. This indicates that the MOO approach can produce good solutions in every run, thanks to a wide exploration of the objective space, which is prevented by a single-objective approach. Additionally, also in this case it is possible to observe different kinds of behaviours evolved with the MOO approach, similarly to what was shown in Section 3 (refer also to [64]). Some of the evolved behaviours are available as supplementary material in S2 Video.

## 5 Case Study: a Strictly Collaborative Task

**Fig 8:**
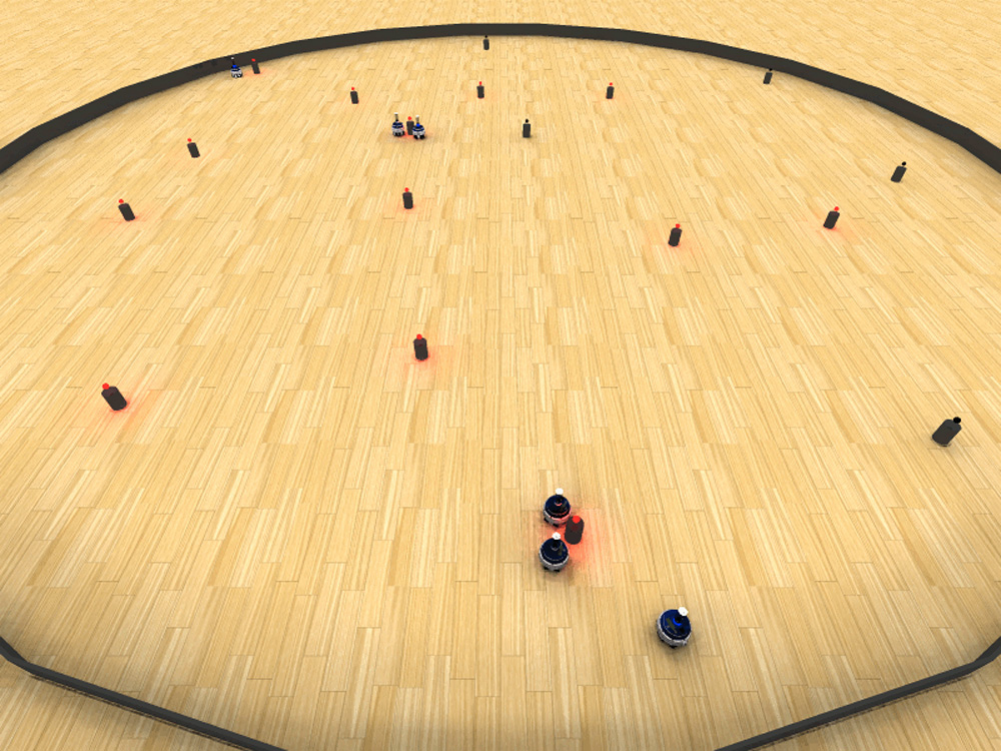
Setup of the strictly collaborative task experiment. The image shows a view of the arena in which both robots and beacons are visible. Some beacons have been already switched off, while it is possible to see that two robots are waiting for collaboration from a third one that is approaching. Experiments are performed in simulation [21]. Some examples of the evolved behaviours for both *n* = 2 and *n* = 3 are available as supplementary material in S3 Video.

This last case study is dedicated to a strictly collaborative task, that is, a task in which collaboration among multiple robots is strictly necessary. In other words, there are no other possibilities than success—when a fruitful collaboration is established—or failure. The notion of strictly collaborative tasks has been introduced by Alcherio Martinoli and collaborators in the context of the “stick-pulling experiment” [23]. In this experiment, robots had to pull a long stick out of the ground, and the collaboration of two robots was necessary as the stick was too long to be extracted by a single robot. They designed a behaviour-based controller and studied the performance of the system in terms of the number of pulled-out sticks after a fixed amount of time. Their proposed solution is based on a timeout mechanism: robots explore the arena and wait for teammates when they encounter a stick to be extracted; if no teammate arrives within a given time interval, a timeout is triggered so that the robot abandons the stick and resumes exploration. By appropriately tuning the waiting time, the performance of the system can be optimised. Inspired by this experiment, we present a strictly collaborative task in which we use beacon lights to be switched off instead of sticks to be pulled out from the ground. Beacons are made by red LEDs positioned over cylindrical obstacles and are perceived by the robots through their omnidirectional camera (see Fig 8). A beacon is switched off automatically when at least *n* robots are close enough at the same time, therefore enforcing the need for collaboration. The details of the experimental setup are available as supplementary material in S1 Text.

The evolution of collective behaviours for strictly collaborative tasks is not trivial. The complexity of such tasks depends on the ratio between the number *M* of objects to be processed (sticks or beacons) and the number *N* of robots in the group, and non-trivial strategies are required when *N*/*M* ≪ 1. In this study, we introduce an additional parameter to control the task complexity, that is, the number of robots *n* required in a single collaboration, which we refer to as the collaboration level. In this way, we can vary the task complexity and observe its bearing on the evolvability of collaborative strategies.

A delicate aspect of strictly collaborative tasks is that the intrinsic measure of performance—the number of pulled-out sticks or switched-off beacons—does not offer much gradient for evolutionary optimisation as long as some form of collaboration is not in place. Indeed, either robots know how to collaborate, and therefore their performance reflects their ability, or they do not, and in this case they would score a null fitness. This means that the evolution of controllers for strictly collaborative tasks may be affected by the bootstrap problem, because it is very likely that randomly generated solutions do not correspond to any collaboration ability. In this section, we demonstrate that it is possible to bypass the bootstrap problem and provide a performance gradient for evolutionary optimisation by exploiting MOO as a process helper through the introduction of some ancillary objective that can guide evolution in the early phases. In Section 5.1, we introduce the main and ancillary objectives for our strictly collaborative task, while the obtained results are presented in Section 5.2.

### 5.1 Main and ancillary objectives in strictly collaborative tasks

As mentioned above, the task we have defined requires that *N* = 6 robots collaborate to switch off *M* = 18 beacons, randomly scattered in a circular arena (radius: 6 m). The beacons are automatically switched off when at least *n* robots are within a radius *r*_*v*_ = 25 cm from the beacon. The intrinsic performance metric for this task corresponds to the fraction of switched-off beacons at the end of a trial:

$$\begin{array}{ccc} S_{c,n} & = & \frac{|B_{0}\left(T\right)|}{M}, \end{array}$$ (10)

$$\begin{array}{ccc} B_{0}\left(t\right) & = & \{b,\;s_{b}\left(t\right)=0,b=1,\ldots ,M\}, \end{array}$$ (11)

where *B*_0_(*t*) represents the set of beacons *b* whose status *s*_*b*_(*t*) is off at time *t*, and *T* is the length of a trial. The state of a beacon is always on (*s*_*b*_ = 1) unless at least *n* robots are close enough at the same time:

$$s_{b}\left(t\right)=0\Leftrightarrow \{\;\begin{array}{c} \exists A^{\prime }\subset A,\left|A^{\prime }\right|=n\\ \exists t^{\prime }\leq t\;\forall i\in A^{\prime }\;d_{i,b}\left(t^{\prime }\right)\leq r_{v} \end{array},$$ (12)

where *A*′ is a subset of cardinality *n* of the set of robots *A*, and *d*_*i*,*b*_(*t*) = ∥**X**_*i*_(*t*) − **X**_*b*_(*t*)∥ is the euclidean distance between robot *i* and beacon *b*. Similarly to [23], 𝓢_*c*,*n*_ represents the collaboration rate within a trial of length *T*. Behaviours in [23] were designed by hand. Here, we use 𝓢_*c*,*n*_ as the fitness function to evolve behaviours in the SOO approach, and we test it with two collaboration levels (i.e., *n* = 2 and *n* = 3) to explore different task complexities.

Single-objective evolution with 𝓢_*c*,*n*_ is affected by the bootstrap problem, as will be shown in Section 5.2. To overcome this problem, we resort to multi-objectivisation by adding an ancillary objective that is somehow related to the task. To this purpose, we note that collaborations can be obtained if robots are capable of visiting multiple beacons during a trial. Indeed, if a robot stops at the first encountered beacon and never leaves, it is very likely that the group will end in a deadlock condition in which all robots are waiting at different beacons and no collaboration takes place. Therefore, a robot must be capable of visiting multiple beacons during a trial. We compute this exploration performance as follows:

$$\begin{array}{ccc} S_{v} & = & \frac{1}{N}\sum_{i}\frac{|V_{i}\left(T\right)|}{M}, \end{array}$$ (13)

$$\begin{array}{ccc} V_{i}\left(t\right) & = & \{b,\;\min_{t^{\prime }\leq t}d_{i,b}\left(t^{\prime }\right)\leq r_{v}\}, \end{array}$$ (14)

where *V*_*i*_(*t*) is the set of beacons visited by robot *i* at time *t*. A beacon *b* is considered visited by robot *i* if the minimum distance achieved from the beacon is less than *r*_*v*_. By visiting multiple beacons, the robots maximise their exploration abilities. However, a robot needs to remain close to a beacon for some time in order to establish a collaboration with its teammates. Therefore, a tradeoff is present between the maximisation of the exploration ability and the waiting time for collaboration. The MOO approach is most suited to explore this tradeoff and eventually provide solutions capable of maximising 𝓢_*c*,*n*_, which remains our main goal.

### 5.2 Results

**Fig 9:**
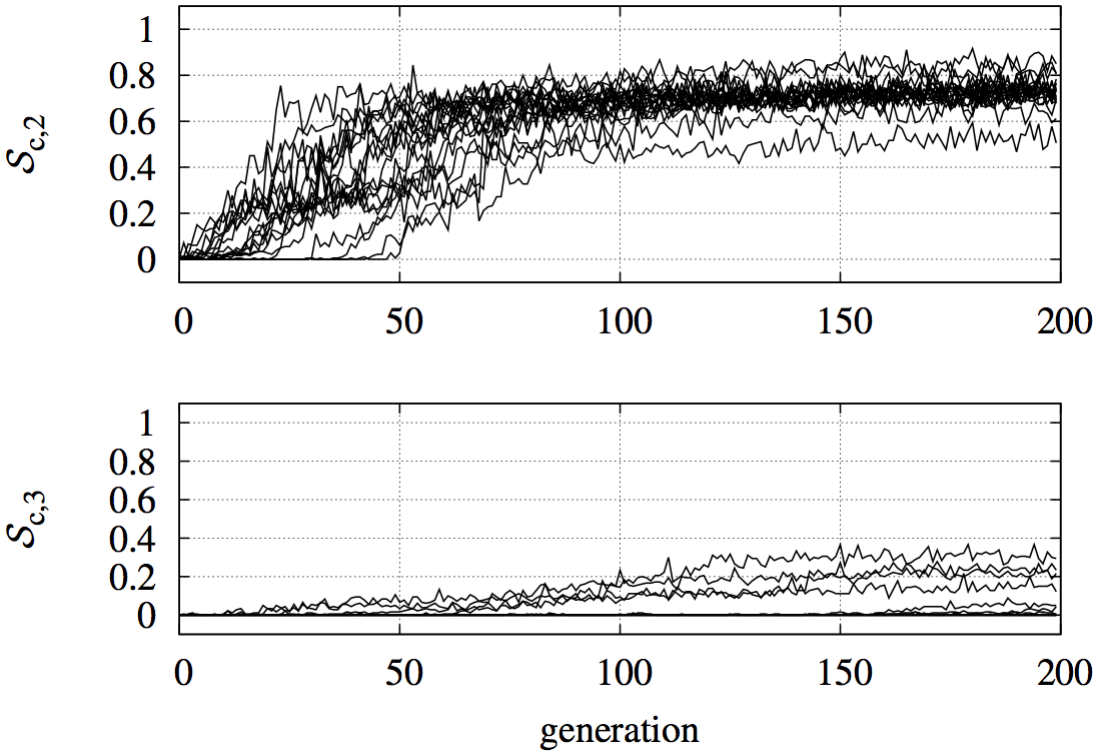
Strictly Collaborative Task. Fitness of the best individual across generation for all evolutionary runs, for *n* = 2 (top) and *n* = 3 (bottom). Note that the maximum fitness achievable with *n* = 3 is lower than with *n* = 2, due to the increased complexity of the task.

As mentioned above, we study two different task complexities by setting the collaboration level to *n* = 2 and *n* = 3. We run single-objective evolution using 𝓢_*c*,*n*_ as fitness function. For each evolutionary run, we first observe the trend of the fitness of the best individual in the population during the evolutionary optimisation, as shown in Fig 9. We notice that the bootstrap problem only mildly affects evolution with *n* = 2. Several evolutionary runs do show a flat fitness surface for many generations, which indicates that the whole population scored a null fitness. However, by random drift, some solutions with non-null fitness appeared and optimisation rapidly started. This indicates that the bootstrap problem exists, but is not too severe. On the contrary, when *n* = 3 the situation is worse, as can be seen in the bottom part of Fig 9. Most of the evolutionary runs suffered of the bootstrap problem, and present a very flat fitness surface. Only a minority of runs were able to discover a suitable strategy that was optimised through the generations. This also indicates that there exist possible solutions to the proposed problem, but they are not easily obtained by a SOO approach.

**Fig 10:**
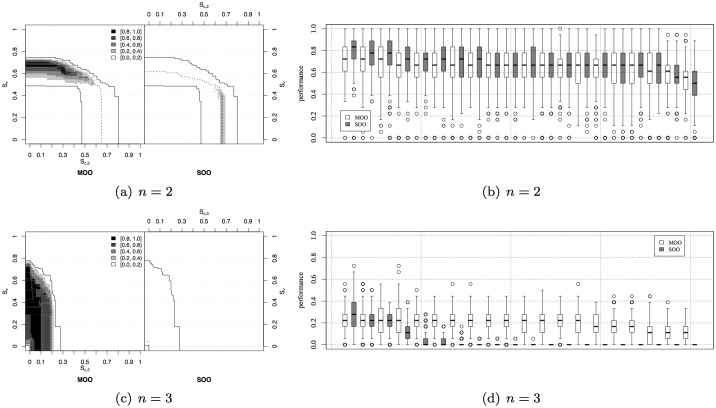
Strictly Collaborative Task. Left: Differences between the EAFs of the MOO and the SOO approaches (see caption of Fig 2 for an explanation of the EAFs difference). Right: performance of the best individuals from the different evolutionary runs with respect to 𝓢_*c*,*n*_, ordered by decreasing median and mean performance.

Once confirmed the existence of the bootstrap problem, in both a mild and severe version, it is worth comparing the performance of the MOO approach with respect to the SOO one. We are interested in studying whether multi-objective evolution is capable of producing solutions as efficient as those evolved in the single-objective case (especially for *n* = 2) and whether the bootstrap problem can be bypassed by systematically evolving good solutions in every evolutionary run (especially for *n* = 3). The results of the comparison for *n* = 2 are shown in Fig 10a and 10b. By looking at the difference between the EAFs of the MOO and SOO approaches (Fig 10a), we can observe that the second objective does not prevent to evolve good quality solutions. Indeed, both approaches similarly attain the regions of the objective space where 𝓢_*c*,2_ is maximised. Additionally, the MOO approach also finds solutions that provide good exploration abilities to the robots by maximising 𝓢_*v*_, which cannot be achieved by the SOO approach. This also confirms the existence of a tradeoff between 𝓢_*c*,*n*_ and 𝓢_*v*_ and therefore supports the usage of a MOO approach instead of a scalarization of the two objectives, which may result in poor solutions, as we have discussed in the previous case studies. The similarity in performance between the MOO and SOO approaches is also confirmed by looking at Fig 10b, which shows the performance of the best evolved controllers for each run computed over 500 different trials. We can therefore conclude that the mild bootstrap problem observed when *n* = 2 can be efficiently overcome by both approaches, and no substantial difference in performance can be observed between the two with respect to 𝓢_*c*,2_.

The situation is completely different when the bootstrap problem becomes severe, as is the case for *n* = 3. The comparison between MOO and SOO shown in Fig 10c and 10d demonstrates that the ancillary objective 𝓢_*v*_ is definitely helpful. Indeed, the EAF differences shown in Fig 10c demonstrate a strong advantage of the MOO approach in every area of the objective space. Similarly, the performance comparison among the best evolved individuals shown in Fig 10d indicates that suitable solutions are obtained in every evolutionary run with MOO, which means that the bootstrap problem is bypassed thanks to the introduction of the second objective. Indeed, 𝓢_*v*_ allows guiding the evolutionary optimisation when there is no fitness gradient, because exploration is a beneficial ability to promote collaborations. In this case, random drift alone is not sufficient to produce good solutions, at least not systematically in every run. We can conclude that MOO is a more reliable approach to produce satisfactory behaviours in every evolutionary run, thanks to the ability to bypass the bootstrap problem.

## 6 Discussions and Conclusions

The studies presented in this paper clearly demonstrate how beneficial can be the use of MOO in an evolutionary robotics context. From the experiments we performed, the overall conclusion is that MOO is the choice to be made whenever no a priori knowledge is available about the problem to be solved, because MOO allows a wider search of the space of possible solutions, provides higher evolvability and removes the constraints introduced by custom-tailored formulations of the fitness function. We do not claim that MOO should be used in any case. Indeed, we also show that single-objective evolution is more powerful when a good fitness function is available a priori, because the search effort is focused on the maximisation of the solution performance in a one-dimensional objective space. Instead, MOO diffuses the search effort to adequately approximate the whole Pareto front in a high-dimensional objective space. Consequently, MOO may require more generations to match the quality of the SOO approach with respect to an a priori fitness function. This explains the slight advantage of SOO over MOO that was detected in certain conditions of the experiments presented in this paper. We predict that this advantage would fade by running the evolutionary algorithms for more generations. However, in evolutionary robotics is often the behaviour that has primacy over the performance score, and we have shown that the MOO approach can produce a varied set of strategies to solve a given problem. A similar variety of strategies could be achieved with the SOO approach by exploring multiple trade-offs through several independent scalarizations, even though this approach has several limitations especially when the Pareto set is concave [24]. However, our experiments have shown that, in the context of evolutionary robotics, the MOO approach produces a better approximation to the Pareto front at a lower computational cost.

Furthermore, the availability of a good fitness function is rather exceptional in evolutionary robotics. It is fair to admit that much of the past research in evolutionary robotics concealed the laborious process of finding the right fitness function and experimental design before obtaining good results. We claim that this burden can be very much alleviated by resorting to MOO, because it disentangles several problematic issues:

- With SOO, it is difficult to find the correct trade-off between multiple behavioural terms that represent the desired behaviour of the robotic system, which may have different and non-comparable scales (e.g., varying non-linearly). Trade-offs are not an issue in MOO, because all of them are naturally explored.
- With SOO, premature convergence to local optima must be carefully dealt with. In MOO, local optima are less problematic because there are several possible evolutionary paths to be exploited. Different evolutionary paths correspond to the emergence of behavioural capabilities that can be attained even at the expense of other capabilities, therefore allowing the evolution of diverse behavioural strategies.
- With SOO, the bootstrap problem can be a severe limitation in the usage of simple fitness functions related to task performance, above all when reward is sporadic or conditioned to the existence of preliminary behavioural capabilities, such as the ability to communicate and collaborate. In MOO, task performance can be optimised exploiting the guidance from ancillary objectives that contribute to the evolution of the behavioural pre-requisites necessary for task achievement.

From an engineering perspective, MOO represents a valid methodology to synthesise a wide set of potential solutions to a given robotics problem. A common criticism to MOO approaches is that eventually a single solution must be selected, therefore part of the design process is anyway arbitrarily driven by the designer. This is actually a feeble argument, because the supposedly arbitrary choice can be made on a limited set of Pareto-optimal solutions and can exploit the available information on the trade-offs they optimise. Additionally, in evolutionary robotics, the detailed analysis of several obtained solutions is common practice even within the SOO approach. The individuals produced during an evolutionary run are evaluated for the ability to generalise to a wide range of environmental conditions, which are loosely sampled during the optimisation process. More than being a generalisation test, this is actually the verification that the evolved solutions meet the design requirements. A similar verification process is necessary also with the MOO approach, but in this case one can limit the analysis to the Pareto set.

To conclude, we strongly believe that the usage of MOO in evolutionary robotics has more advantages than drawbacks, and should therefore be promoted whenever the need of multiple objectives arises. Future work should be focused on collecting more results on the advantages of MOO over SOO in several test-cases, therefore leading to the establishment of a multi-objective methodology for evolutionary robotics. We tested a relatively simple MOO algorithm that differs only from its SOO counterpart on the selection step, and a more detailed experimental analysis would be required to establish good guidelines for the choice of the MOO algorithm and its parameters. Additionally, the interaction of MOO with other design choices providing selective pressures must be considered [3]. For instance, important design choices correspond to the definition of the robot configuration or of the genotype-to-phenotype mapping [7, 64]. Some aspects of these choices can be delegated to evolutionary optimisation in the form of additional objectives, and studies going in this direction have been mentioned in Section 2. Obviously, adding an arbitrary number of objectives would hinder the quality of the obtained results simply by the fact that increasing the number of conflicting objectives leads to a higher fraction of the objective space becoming Pareto-optimal, and the difficulty then lies on choosing a solution rather than on finding it [56, 65]. However, a more systematic study of the different design choices in a multi-objective perspective is required.

Finally, some effort must be dedicated to extend the methodology to realistic, application-driven scenarios. In this respect, the possibility to address also the simulation-to-reality gap within a multi-objective approach [35] represents a further demonstration of the potentials of MOO for pushing forward evolutionary robotics research. Another under-explored aspect is interactive MOO [19, 20]. Some research on interactive evolution in robotics has been performed to date [49, 50, 66–68]. However, this is a very human-intensive task that requires robotic designers to explicitly select the genotypes that would reproduce. By contrast, interactive MOO allows designers to progressively articulate their preferences with respect to the evolving behaviour by examining (some) Pareto-optimal solutions produced while running a multi-objective evolutionary algorithm, in order to focus the search in the most interesting regions of the objective space. Hence, it could be possible to include within the design process the intrinsic subjectivity of the evolution of robotics behaviours.

## Supporting Information

S1 Text Experimental methods. The document describes in detail the experimental procedures for comparing MOO and SOO approaches. Additionally, the experimental setup is fully described with details about the robots, the simulation framework and the evolutionary experiments.
(PDF)

S1 Video Navigation experiment. This video refers to the first case study on navigation in a maze, and shows the differences between the wall-following and quick-avoidance strategies. Size: 2.8 MB, MPEG-4 (H.264 codec).
(MP4)

S2 Video Flocking experiment. This video refers to the flocking case study, and shows different types of flocking behaviour evolved. Size: 4.4 MB, MPEG-4 (H.264 codec).
(MP4)

S3 Video Strictly collaborative task. This video refers to the case study on the strictly collaborative task, and shows the evolved behaviour for two different collaboration levels. Size: 15.7 MB, MPEG-4 (H.264 codec).
(MP4)

## References

[1] Nolfi S, Floreano D. Evolutionary Robotics: The Biology, Intelligence, and Technology of Self-organizing Machines. MIT Press, Cambridge, MA; 2000.

[2] Floreano D, Keller L. Evolution of Adaptive Behaviour in Robots by Means of Darwinian Selection. PLoS Biology. 2010;8(1):e1000292 10.1371/journal.pbio.1000292 20126252 PMC2811146

[3] Doncieux S, Mouret JB. Beyond black-box optimization: a review of selective pressures for evolutionary robotics. Evolutionary Intelligence. 2014;7(2):71–93. 10.1007/s12065-014-0110-x

[4] Doncieux S, Bredeche N, Mouret JB, Eiben AEG. Evolutionary Robotics: What, Why, and Where to. Frontiers in Robotics and AI. 2015;2(4):1–18.

[5] Pfeifer R, Lungarella M, Iida F. Self-Organization, Embodiment, and Biologically Inspired Robotics. Science. 2007;318(5853):1088–1093. 10.1126/science.1145803 18006736

[6] Lipson H. Evolutionary Design and Open-Ended Design Automation In: Bar-Cohen Y, editor. Biomimetics: Biologically Inspired Technologies. Taylor & Francis/CRC Press, Boca Raton, FL; 2005 p. 129–155.

[7] Trianni V, Nolfi S. Engineering the evolution of self-organizing behaviors in swarm robotics: A case study. Artificial Life. 2011;17(3):183–202. 10.1162/artl_a_00031 21554112

[8] Lehman J, Stanley KO. Abandoning Objectives: Evolution Through the Search for Novelty Alone. Evolutionary computation. 2011;19(2):189–223. 10.1162/EVCO_a_00025 20868264

[9] Mouret JB, Doncieux S. Encouraging behavioral diversity in evolutionary robotics: An empirical study. Evolutionary computation. 2012;20(1):91–133. 10.1162/EVCO_a_00048 21838553

[10] Floreano D, Urzelai J. Evolutionary robots with on-line self-organization and behavioral fitness. Neural Networks. 2000;13(4-5):431–443. 10.1016/S0893-6080(00)00032-0 10946391

[11] Nelson AL, Barlow GJ, Doitsidis L. Fitness functions in evolutionary robotics: A survey and analysis. Robotics and Autonomous Systems. 2009;57(4):345–370. 10.1016/j.robot.2008.09.009

[12] Wagner GP, Altenberg L. Perspective: complex adaptations and the evolution of evolvability. Evolution. 1996;50(3):967–976. 10.2307/2410639 28565291

[13] Mouret JB, Doncieux S. Incremental Evolution of Animats’ Behaviors as a Multi-objective Optimization In: Asada M, Hallam JC, Meyer JA, Tani J, editors. From animals to animats 10: Proceedings of the 10th International Conference on Simulation of Adaptive Behavior (SAB’08). Berlin, Germany: Springer Verlag; 2008 p. 210–219.

[14] Mouret JB, Doncieux S. Overcoming the bootstrap problem in evolutionary robotics using behavioral diversity In: Proceedings of the 2009 IEEE Congress on Evolutionary Computation (CEC 2009). Piscataway, NJ: IEEE Press; 2009 p. 1161–1168.

[15] Coello Coello CA, Lamont GB, Van Veldhuizen DA. Evolutionary Algorithms for Solving Multi-Objective Problems. Springer, New York, NY; 2007.

[16] Deb K. Multi-Objective Optimization Using Evolutionary Algorithms. Chichester, UK: Wiley; 2001.

[17] Marler RT, Arora JS. Survey of multi-objective optimization methods for engineering. Structural and Multidisciplinary Optimization. 2004 4;26(6):369–395. 10.1007/s00158-003-0368-6

[18] Handl J, Knowles JD. Modes of Problem Solving with Multiple Objectives: Implications for Interpreting the Pareto Set and for Decision Making In: Knowles JD, Corne D, Deb K, Chair DR, editors. Multiobjective Problem Solving from Nature. Springer; 2008 p. 131–151.

[19] Branke J, Deb K, Miettinen K, Słowiński R, editors. Multi-objective Optimization: Interactive and Evolutionary Approaches vol. 5252 of LNCS. Springer; 2008.

[20] Jaszkiewicz A, Branke J. Interactive Multiobjective Evolutionary Algorithms In: Branke J, Deb K, Miettinen K, Słowiński R, editors. Multi-objective Optimization: Interactive and Evolutionary Approaches. vol. 5252 of LNCS. Springer; 2008 p. 179–193.

[21] Pinciroli C, Trianni V, O’Grady R, Pini G, Brutschy A, Brambilla M, et al ARGoS: A Modular, Parallel, Multi-Engine Simulator for Multi-Robot Systems. Swarm Intelligence. 2012;6(4):271–295. 10.1007/s11721-012-0072-5

[22] Floreano D, Mondada F. Automatic Creation of an Autonomous Agent: Genetic Evolution of a Neural Network Driven Robot In: From Animals to Animats 3: Proceedings of the 3rd International Conference on Simulation of Adaptive Behavior (SAB’94). Cambridge, MA: MIT Press; 1994 p. 421–430.

[23] Ijspeert AJ, Martinoli A, Billard A, Gambardella LM. Collaboration Through the Exploitation of Local Interactions in Autonomous Collective Robotics: The Stick Pulling Experiment. Autonomous Robots. 2001;11(2):149–171. 10.1023/A:1011227210047

[24] Das I, Dennis JE. A closer look at drawbacks of minimizing weighted sums of objectives for Pareto set generation in multicriteria optimization problems. Structural Optimization. 1997;14(1):63–69. 10.1007/BF01197559

[25] Teo J, Abbass HA. Automatic generation of controllers for embodied legged organisms: A Pareto evolutionary multi-objective approach. Evolutionary Computation. 2004;12(3):355–394. 10.1162/1063656041774974 15355605

[26] Teo J, Abbass HA. Multiobjectivity and complexity in embodied cognition. IEEE Transactions on Evolutionary Computation. 2005;9(4):337–360. 10.1109/TEVC.2005.846902

[27] Agapitos A, Togelius J, Lucas SM. Multiobjective techniques for the use of state in genetic programming applied to simulated car racing In: Proceedings of the 2007 IEEE Congress on Evolutionary Computation (CEC 2007). Piscataway, NJ: IEEE Press; 2007 p. 1562–1569.

[28] Bongard JC. The utility of evolving simulated robot morphology increases with task complexity for object manipulation. Artificial Life. 2010;16(3):201–223. 10.1162/artl.2010.Bongard.024 20059328

[29] Bongard JC. Innocent Until Proven Guilty: Reducing Robot Shaping From Polynomial to Linear Time. IEEE Transactions on Evolutionary Computation. 2011;15(4):571–585. 10.1109/TEVC.2010.2096540

[30] Auerbach JE, Bongard JC. Environmental Influence on the Evolution of Morphological Complexity in Machines. PLoS Computational Biology. 2014;10(1):e1003399 10.1371/journal.pcbi.1003399 24391483 PMC3879106

[31] de Margerie E, Mouret JB, Doncieux S, Meyer JA. Artificial evolution of the morphology and kinematics in a flapping-wing mini-UAV. Bioinspiration & Biomimetics. 2007;2(4):65–82. 10.1088/1748-3182/2/4/002 18037730

[32] Mouret JB, Doncieux S, Meyer JA. Incremental Evolution of Target-Following Neuro-controllers for Flapping-Wing Animats In: Nolfi S, Baldassarre G, Calabretta R, Hallam JT, Marocco D, Meyer JA, et al, editors. From Animals to Animats 9: Proceedings of the 9th International Conference on Simulation of Adaptive Behavior (SAB’06). Berlin, Germany: Springer Verlag; 2006 p. 606–618.

[33] Terekhov AV, Mouret JB, Grand C. Stochastic optimization of a neural network-based controller for aggressive maneuvers on loose surfaces In: Proceedings of the 2010 IEEE/RSJ International Conference on Intelligent Robots and Systems (IROS 2010). Piscataway, NJ: IEEE Press; 2010 p. 4782–4787.

[34] Terekhov AV, Mouret JB, Grand C. Stochastic optimization of a chain sliding mode controller for the mobile robot maneuvering In: Proceedings of the 2011 IEEE/RSJ International Conference on Intelligent Robots and Systems (IROS 2011). Piscataway, NJ: IEEE Press; 2011 p. 4360–4365.

[35] Koos S, Mouret JB, Doncieux S. The Transferability Approach: Crossing the Reality Gap in Evolutionary Robotics. IEEE Transactions on Evolutionary Computation. 2013;17(1):122–145. 10.1109/TEVC.2012.2185849

[36] Lehman J, Stanley KO. Evolving a diversity of creatures through novelty search and local competition In: Proceedings of the 13th International Conference on Genetic and Evolutionary Computation (GECCO’11). New York, NY: ACM Press; 2011 p. 211–218.

[37] Lehman J, Risi S, D’Ambrosio D, Stanley KO. Encouraging reactivity to create robust machines. Adaptive Behavior. 2013;21(6):484–500. 10.1177/1059712313487390

[38] Koos S, Cully A, Mouret JB. Fast damage recovery in robotics with the T-resilience algorithm. The International Journal of Robotics Research. 2013;32(14):1700–1723. 10.1177/0278364913499192

[39] Pinville T, Koos S, Mouret JB, Doncieux S. How to promote generalisation in evolutionary robotics: the ProGAb approach In: Proceedings of the 13th International Conference on Genetic and Evolutionary Computation (GECCO’11). New York, NY: ACM Press; 2011 p. 259–266.

[40] Ollion C, Pinville T, Doncieux S. With a little help from selection pressures: evolution of memory in robot controllers In: Artificial Life 13: International Conference on the Simulation and Synthesis of Living Systems. Cambridge, MA: MIT Press; 2012 p. 407–414.

[41] Capi G. Multiobjective evolution of neural controllers and task complexity. IEEE Transactions on Robotics. 2007;23(6):1225–1234. 10.1109/TRO.2007.910773

[42] Barlow GJ, Oh CK, Grant E. Incremental evolution of autonomous controllers for unmanned aerial vehicles using multi-objective genetic programming In: Proceedings of the IEEE Conference on Cybernetics and Intelligent Systems. vol. 2 Piscataway, NJ: IEEE Press; 2004 p. 689–694.

[43] Moshaiov A, Ashram A. Multi-objective evolution of robot neuro-controllers In: Proceedings of the 2009 IEEE Congress on Evolutionary Computation (CEC’09). Piscataway, NJ: IEEE Press; 2009 p. 1093–1100.

[44] Moshaiov A, Abramovich O. Is MO-CMA-ES superior to NSGA-II for the evolution of multi-objective neuro-controllers? In: Proceedings of the 2014 IEEE Congress on Evolutionary Computation (CEC 2014). Piscataway, NJ: IEEE Press; 2014 p. 2809–2816.

[45] Oliveira M, Matos V, Santos CP, Costa L. Multi-objective parameter CPG optimization for gait generation of a biped robot In: Proceedings of the 2013 IEEE International Conference on Robotics and Automation (ICRA 201). Piscataway, NJ: IEEE Press; 2013 p. 3130–3135.

[46] Oliveira M, Doncieux S, Mouret JB, Peixoto Santos C. Optimization of humanoid walking controller: Crossing the reality gap In: Proceedings of the 13th IEEE-RAS International Conference on Humanoid Robots (Humanoids 2013). Piscataway, NJ: IEEE Press; 2013 p. 106–111.

[47] Herrera Ortiz JA, Rodríguez-Vázquez K, Padilla Castañeda MA, Arámbula Cosío F. Autonomous robot navigation based on the evolutionary multi-objective optimization of potential fields. Engineering Optimization. 2013;45(1):19–43. 10.1080/0305215X.2012.658781

[48] Doncieux S. Transfer learning for direct policy search: A reward shaping approach In: Proceedings of the 2013 IEEE Third Joint International Conference on Development and Learning and Epigenetic Robotics (ICDL 2013). Piscataway, NJ: IEEE Press; 2013 p. 1–6.

[49] Bongard JC, Hornby GS. Combining fitness-based search and user modeling in evolutionary robotics In: Proceedings of the 15th International Conference on Genetic and Evolutionary Computation (GECCO’13). New York, NY: ACM Press; 2013 p. 159–166.

[50] Bernatskiy A, Hornby GS, Bongard JC. Improving Robot Behavior Optimization by Combining User Preferences In: Sayama H, Rieffel J, Risi S, Doursat R, Lipson H, editors. Artificial Life 14: International Conference on the Synthesis and Simulation of Living Systems. Cambridge, MA: MIT Press; 2014 p. 726–733.

[51] Mouret JB. Novelty-Based Multiobjectivization In: Doncieux S, Bredeche N, Mouret JB, editors. New Horizons in Evolutionary Robotics. Berlin, Germany: Springer Verlag; 2011 p. 139–154.

[52] Lehman J, Stanley KO, Miikkulainen R. Effective diversity maintenance in deceptive domains In: Proceedings of the 15th International Conference on Genetic and Evolutionary Computation (GECCO’13). New York, NY: ACM Press; 2013 p. 215–222.

[53] Bonani M, Longchamp V, Magnenat S, Rétornaz P, Burnier D, Roulet G, et al The MarXbot, a Miniature Mobile Robot Opening new Perspectives for the Collective-robotic Research In: Proceedings of the 2010 IEEE/RSJ International Conference on Intelligent Robots and Systems (IROS 2010). Piscataway, NJ: IEEE Press; 2010 p. 4187–4193.

[54] Zitzler E, Thiele L. Multiobjective Optimization using evolutionary algorithms—A comparative case study In: Eiben AE, Bäck T, Schoenauer M, Schwefel HP, editors. Parallel Problem Solving from Nature, PPSN V. vol. 1498 of LNCS. Springer; 1998 p. 292–301.

[55] Beume N, Naujoks B, Emmerich M. SMS-EMOA: Multiobjective selection based on dominated hypervolume. European Journal of Operational Research. 2007;181(3):1653–1669. 10.1016/j.ejor.2006.08.008

[56] Bader J, Zitzler E. HypE: An Algorithm for Fast Hypervolume-Based Many-Objective Optimization. Evolutionary Computation. 2011;19(1):45–76. 10.1162/EVCO_a_00009 20649424

[57] Grunert da Fonseca V, Fonseca CM, Hall AO. Inferential Performance Assessment of Stochastic Optimisers and the Attainment Function In: Zitzler E, Deb K, Thiele L, Coello Coello CA, Corne D, editors. Evolutionary Multi-criterion Optimization, EMO 2001. vol. 1993 of LNCS. Springer; 2001 p. 213–225.

[58] Grunert da Fonseca V, Fonseca CM. The Attainment-Function Approach to Stochastic Multiobjective Optimizer Assessment and Comparison In: Bartz-Beielstein T, Chiarandini M, Paquete L, Preuss M, editors. Experimental Methods for the Analysis of Optimization Algorithms. Berlin, Germany: Springer; 2010 p. 103–130.

[59] López-Ibáñez M, Paquete L, Stützle T. Exploratory Analysis of Stochastic Local Search Algorithms in Biobjective Optimization In: Bartz-Beielstein T, Chiarandini M, Paquete L, Preuss M, editors. Experimental Methods for the Analysis of Optimization Algorithms. Berlin, Germany: Springer; 2010 p. 209–222.

[60] Reynolds CW. Flocks, herds and schools: A distributed behavioral model In: Proceedings of the 14th annual conference on Computer graphics and interactive techniques. SIGGRAPH’87. New York, NY: ACM Press; 1987 p. 25–34.

[61] Vicsek T, Zafeiris A. Collective motion. Physics Reports. 2012;517(3-4):71–140. 10.1016/j.physrep.2012.03.004

[62] Quinn M, Smith L, Mayley G, Husbands P. Evolving controllers for a homogeneous system of physical robots: Structured cooperation with minimal sensors. Philosophical Transactions of the Royal Society of London, Series A: Mathematical, Physical and Engineering Sciences. 2003;361(1811):2321–2343. 10.1098/rsta.2003.1258 14599322

[63] Baldassarre G, Nolfi S, Parisi D. Evolving Mobile Robots Able to Display Collective Behaviors. Artificial Life. 2013;9(3):255–267. 10.1162/106454603322392460 14556687

[64] Fehérvári I, Trianni V, Elmenreich W. On the Effects of the Robot Configuration on Evolving Coordinated Motion Behaviors In: Proceedings of the 2013 IEEE Congress on Evolutionary Computation (CEC 2013). Piscataway, NJ: IEEE Press; 2013 p. 1209–1216.

[65] Ishibuchi H, Tsukamoto N, Nojima Y. Evolutionary many-objective optimization: A short review In: Proceedings of the 2008 Congress on Evolutionary Computation (CEC 2008). Piscataway, NJ: IEEE Press; 2008 p. 2419–2426.

[66] Gruau F, Quatramaran K. Cellular encoding for interactive evolutionary robotics In: Husbands P, Harvey I, editors. Proceedings of the 4th European Conference on Artificial Life (ECAL 1997). Cambridge, MA: MIT Press; 1997 p. 368–377.

[67] Takagi H. Interactive evolutionary computation: fusion of the capabilities of EC optimization and human evaluation. Proceedings of the IEEE. 2001;89(9):1275–1296. 10.1109/5.949485

[68] Miglino O, Gigliotta O, Ponticorvo M, Lund HH. Human breeders for evolving robots. Artificial Life and Robotics. 2008;13(1):1–4. 10.1007/s10015-008-0503-y

