# Correction: Advantages of Task-Specific Multi-Objective Optimisation in Evolutionary Robotics

**DOI:** 10.1371/journal.pone.0140056

**Published:** 2015-10-02

**Authors:** Vito Trianni, Manuel López-Ibáñez

The following information is missing from the Funding section: Manuel López-Ibáñez acknowledges support from the Belgian F.R.S.-FNRS, of which he is a postdoctoral researcher.

S1 Text has been corrected for improved readability. Please view the correct S1 Text below.

## Supporting Information

S1 Text Experimental methods. The document describes in detail the experimental procedures for comparing MOO and SOO approaches. Additionally, the experimental setup is fully described with details about the robots, the simulation framework and the evolutionary experiments.
(PDF)

## References

[1] Trianni V, López-Ibáñez M (2015) Advantages of Task-Specific Multi-Objective Optimisation in Evolutionary Robotics. PLoS ONE 10(8): e0136406 doi:10.1371/journal.pone.0136406 26295151 10.1371/journal.pone.0136406 PMC4546428

