# Supplementary material for: Advantages of Task-Specific Multi-Objective Optimisation in Evolutionary Robotics
Source: PLoS One. 2015 Aug 21;10(8):e0136406. doi: 10.1371/journal.pone.0136406 (PMC4546428; doi:10.1371/journal.pone.0136406)
Supplement: S1 Text — The document describes in detail the experimental procedures for comparing MOO and SOO approaches. Additionally, the experimental setup is fully described with details about the robots, the simulation framework and the evolutionary experiments. (PDF) [file pone.0136406.s001.pdf]

Advantages of multi-objective optimisation in evolutionary robotics: survey and case studies - Experimental methods

Vito Trianni[1,*], Manuel López-Ibáñez[2]

1 Institute of Cognitive Sciences and Technologies (ISTC), National Research Council (CNR), Rome, Italy.
2 IRIDIA, CoDE, Université Libre de Bruxelles (ULB), Brussels, Belgium

*** vito.trianni@istc.cnr.it**

1 Basic elements of MOO

Many real-world optimization problems are evaluated in terms of multiple, often conflicting criteria or objective functions. When there is no a priori information about the importance of each objective, the solutions to such a multi-objective optimization (MOO) problem are usually compared in terms of Pareto dominance [1, 2]: A solution *dominates* another one if the former is not worse than the latter in all objectives and strictly better in at least one. The goal when tackling such a MOO problem is to find, or approximate as well as possible, the set of all solutions whose image in the objective space is not dominated by any other feasible solution. This set is called the Pareto set and its image is called the Pareto front.

Computing the Pareto front is often intractable in practice and heuristic methods are necessary to generate a high-quality approximation [3]. Among the heuristic methods, multi-objective evolutionary algorithms (MOEAs) have achieved a considerable success and we refer the reader to the many textbooks available on the subject for a detailed introduction [1, 2].

2 Comparison of MOO and SOO approaches

The experimental methodology followed in this paper has been defined to contrast the MOO approach to evolutionary robotics with a standard SOO approach. To this purpose, we have designed an evolutionary algorithm loosely inspired from a canonical (μ, λ)-ES, which is characterised by rank-based selection [4]. Between the single- and multi-objective approaches, the algorithm differs only in the ranking of the population before selection. In the single-objective case, the ranking is based on the genotype fitness, the best individual being the one with the highest fitness score (assuming a maximisation problem). In the multi-objective case, we have chosen to rank the population according to the *hypervolume measure*, which gives a higher rank to those solutions that contribute more to the hypervolume defined by the Pareto front [5]. The usage of the hypervolume for ranking solutions in evolutionary optimisation is currently used in state-of-the-art multi-objective evolutionary algorithms like SMS-EMOA [6] and HypE [7]. Being interested in a fair comparison between SOO and MOO, we do not exploit state-of-the-art MOEAs which may implement techniques that cannot be translated to the SOO case. Instead, we provide an implementation which differs minimally between the two experimental conditions, in order to isolate the contribution given by ranking the population according to a single or to multiple objectives.

Table 1. The evolutionary algorithm used, loosely inspired by a canonical (μ, λ)-ES. Note that the differences between the MOO and the SOO version are solely in step 7 for the computation of a single or of multiple objectives, and in step 8 for the population ranking. The algorithm is implemented using the Shark machine learning library [8], which also provides a readily available procedure for ranking based on the hypervolume measure.

1: **procedure** EVOLUTIONARY ALGORITHM
2:     **for** $0 \le i < \lambda$ **do** ▷ population initialisation
3:         Initialise($\mathbf{p}_i$)
4:     **end for**
5:     $\mathcal{P} \leftarrow \{\mathbf{p}_i, i \in [0, \lambda)\}$
6:     **for** $0 \le g < G$ **do** ▷ repeat for G generations
7:         Evaluate($\mathcal{P}$)
8:         Rank($\mathcal{P}$)
9:         **for** $0 \le i < \eta$ **do** ▷ η-elitism
10:             $\hat{\mathbf{p}}_i \leftarrow \mathbf{p}_i$
11:         **end for**
12:         **for** $\eta \le i < \lambda$ **do** ▷ rank-based μ-selection
13:             $j \leftarrow (i - \eta) \mod \mu$
14:             $\hat{\mathbf{p}}_i \leftarrow$ Mutation($\mathbf{p}_j$)
15:         **end for**
16:         $\mathcal{P} \leftarrow \{\hat{\mathbf{p}}_i, i \in [0, \lambda)\}$
17:     **end for**
18: **end procedure**

The evolutionary algorithm used is detailed in Table 1. Individuals in the population are real-valued vectors $\mathbf{p}_i$. Each element of the vector is initialised by sampling a uniform distribution in the range $[-p_m, p_m]$. The main evolutionary loop proceeds by first evaluating the whole population using one or more objectives. Then, the population is ranked from the best to the worst individual, and undergoes selection and reproduction. We employ an elitist strategy, by copying the best η individuals unchanged in the offspring population. The remainder of the population is generated from a reproductive pool selected as the best μ individuals, by applying only a mutation operator. Mutation is applied by adding a random value drawn from a gaussian distribution $N(0, \sigma)$ to each element of the genotype, possibly cutting off the value to constrain it within the interval $[-p_m, p_m]$. The process is iterated for a maximum of G generations, and results are collected from the final population. See Table 2 for a summary of the algorithm parameters and the value adopted in the experiments.

Performance evaluation is performed in simulation using ARGoS [9], a multi-engine simulator of swarm robotics systems. Because performance evaluation in robotics is dependent on the initial conditions and is influenced by random noise in sensors and actuators, averaging over multiple repetitions (trials) is necessary. For each genotype, we performed $K = 10$ trials initialised with a different seed of the random number generator. The performance of the genotype is the average over all trials, both in the single- and in the multi-objective case. In the latter case, the average is performed separately for the different objectives. In each generation, all genotypes in the population are evaluated against the same K initial conditions, in order to ensure a fair comparison for ranking. Across generations, the K initial conditions are randomly chosen and any possible information available on previous performance is discarded (e.g., for the elite).

For each experimental condition, we performed $R = 20$ different evolutionary runs,

Table 2. Value of the parameters used for the evolutionary algorithm

Parameter	Description	Value
p_m	max parameter absolute value	5
λ	population size	100
η	elite size	25
μ	reproductive pool	25
σ	standard deviation of mutations	$0.2 \cdot p_m$
G	maximum generation number	200
K	repetitions for evaluation	10
$\hat{K}$	repetitions for post-evaluation	500
R	number of runs per experimental condition	20

each starting with a randomly initialised population. The R initial populations are identical across different experimental conditions, to further ensure comparability of the obtained results. For each evolutionary run, we retain only the individuals of the last generation, and we re-evaluate their performance averaging over $\hat{K} = 500$ trials to obtain a bias-free assessment. All the $\hat{K}$ trials (across evolutionary runs and experimental conditions) are performed with the same initial conditions obtained with $\hat{K}$ different seeds of the random number generator. The performance gained in these tests is exploited for comparison. In each evolutionary run, the solutions we consider are either the ones with the highest average performance when accounting for a single objective, or the ones that belong to the (approximate) Pareto front when accounting for multiple objectives.

The SOO and MOO approaches require comparable computational resources in the experiments presented here because evolutionary runs using either the SOO or the MOO approach always execute the same number of objective function evaluations. While the ranking of the population is more computationally expensive in the case of the MOO approach, especially for large number of objectives, this difference is negligible with respect to the time required by a single evaluation of the objective functions in our experiments.

3 Empirical Attainment Functions

Different experimental conditions are compared by means of graphical exploration techniques [10] based on the *empirical attainment function* (EAF). The attainment function [11, 12] gives the probability of a single run of an optimiser *attaining* (dominate or equal) a specific point in the objective space. In most practical cases, such attainment function is unknowable, but it can be empirically estimated from several independent runs of an optimiser. Such an estimation of the attainment function is called the EAF. The EAF statistically summarises the location and variability of the output of stochastic multi-objective optimisers, in an analogous way as the empirical cumulative distribution function in univariate statistics. For instance, those points (x, y) for which $\mathrm{EAF}(x, y) \geq 0.5$ are attained in at least 50% of the runs of the optimiser. We can summarise the EAF by plotting the border of the region attained by at least k% of the runs, called k%-attainment surfaces. For example, Fig. 7 in the main text shows the attainment surfaces with values: 0% (best), 25%, 50% (median), 75% and 100% (worst). These surfaces denote the transition of the EAF to a specific value ($k/100$), that is, these lines correspond to the points in the objective space where the EAF becomes $\geq k/100$. Any point between the k_1% and k_2% attainment surfaces has a value of $\frac{k_1}{100} \leq \mathrm{EAF}(x, y) < \frac{k_2}{100}$. By examining the attainment surfaces of an optimiser (e.g., the plots in Fig. 7 in the main text), we can visualize the expected location and variability

of its output.

A more direct comparison of two optimisers (or experimental conditions) is achieved by plotting the differences between their respective EAFs (see Fig. 6a in the main text for the difference between the EAFs shown in Fig. 7 in the main text). Each side of the plot shows the EAF differences in favour of each optimiser, that is, the points of the objective space where one optimiser has a higher probability than the other of attaining that particular point in a single run. The grey-level gives the magnitude of the difference: darker colours indicate larger differences. For example, a black point on the left indicates that the difference between the EAF of the left optimiser minus the EAF of the right optimiser is at least 0.8, that is, the left optimiser has attained that point in at least 80% more runs than the right one. The solid lines shown in the plot are equal on both sides and correspond to the grand-worst and grand-best attainment surfaces, that is, the best points (in terms of dominance) that are attained, respectively, by all runs of both optimisers and by at least one run of either optimiser. Any EAF differences between the optimisers is located between these two lines. The dashed lines, which are different on each side, denote the 50% attainment surface of the optimiser shown in that side.

4 Experimental Setup

4.1 The marXbot robot and the simulation model

The simulated robot closely models the *marXbot* robotic platform [13], a small wheeled autonomous robot specifically designed for single as well as collective robotics experimentation. The robot has a roughly cylindrical body, and is characterised by tracks and wheels that provide a differential drive motion. It features several sensory and communication devices, including IR proximity sensors for obstacle detection, two cameras (an omni-directional one and a switchable front/ceiling camera), an IR distance scanner, a range-and-bearing communication module to detect and exchange short messages with neighbours, coloured LEDs around the robot body and a powerful RGB beacon placed below the omni-directional camera.

Simulation of the marXbot robot exploits the ARGoS simulator [9]. For the presented experiments, the robot is roughly modelled in two dimensions as a circle, and motion is computed through kinematic equations. This ensures fast simulations even with relatively large groups of robots. Sensors and actuators have been modelled and calibrated to match the features of the physical robot. Random noise is added to every sensor and actuator in order to provide robustness to the synthesised solutions.

4.2 The robot controller

In all experimental studies presented in the paper, robots are controlled by a simple feed-forward neural network whose parameters are optimised by the evolutionary algorithm. Path planning or trajectory tracking strategies are not used. The navigation strategy exploited by the robots is a reactive one, as follows from the usage of a feed-forward neural controller without internal states, which directly maps the sensory inputs to the motor outputs:

$$O_j = z\left(\sum_i w_{ij} I_i + \beta_j\right), \qquad z(x) = \frac{1}{1+e^{-x}}, \tag{1}$$

where $w_{ij} \in [-p_m, p_m]$ is the connection weight between the sensory input I_i and the motor output O_j, and $\beta_j \in [-p_m, p_m]$ is a bias term. Connection weights w_{ij} and bias

terms β_j are the genetically encoded parameters that are subject to artificial evolution. Whenever necessary, the input from sensors are normalised by linearly scaling the readings in the $[0, 1]$ interval before being fed to the neural network. Similarly, the outputs of the network are scaled from the interval $[0, 1]$ to the range of values of the specific actuator.

4.3 Navigation in a maze

For the maze navigation task, we employ a single robot that has to detect walls and obstacles and move along the corridor of a looping maze. We employ only the 24 IR proximity sensors uniformly distributed around the robot body. The readings from the 24 proximity sensors are linearly scaled in the $[0, 1]$ interval, and directly used as inputs for the neural network. The 2 outputs of the neural network are linearly scaled in the range $[-\omega_m, \omega_m]$ with $\omega_m \approx 0.34\ \mathrm{s}^{-1}$, and used to control the angular speed of the two wheels. Therefore we have 24 inputs, 2 outputs and 2 bias terms, resulting in $24 \cdot 2 + 2 = 50$ genes per genotype.

Each evaluation trial is performed by initialising the robot in the looping maze with a random position and orientation. The initial position is chosen close to the centre of the corridor and randomly varying distance from the centre and orientation. Each trial lasts $T = 120$ s and the robot performs a control step every 100 ms.

4.4 Flocking

Flocking is studied with groups of $N = 10$ robots. Each robot is equipped with a minimal set of sensors and actuators, which is however sufficient for displaying a flocking behaviour. The robots use their LEDs to display a left-right pattern that provides some information on their heading. In particular, two red LEDs on the left and two blue LEDs on the right are activated during the whole evaluation trial. These LEDs can be perceived by the omnidirectional camera of neighbouring robots up to the distance of 1 m. Additionally, IR proximity sensors are exploited for close-range collision avoidance.

To provide the inputs to the neural network and to reduce their number, we pre-process both the camera image and the IR proximity sensors as follows. The acquired image is processed to extract a list of red/blue colour blobs $c \in \{r, b\}$ with their distance $\rho_{c,i}$ and angle $\theta_{c,i}$, resulting in a vector $\mathbf{v}_{c,i}(\rho_{c,i}, \theta_{c,i})$ for each detected blob (in polar coordinates). Similarly, for each IR proximity sensor $i = 1, \ldots, 24$ we build a two-dimensional vector $\mathbf{v}_{p,i}(\rho_{p,i}, \theta_{p,i})$ in polar coordinates, where $\rho_{p,i}$ corresponds to the sensor reading, and $\theta_{p,i}$ corresponds to the sensor bearing.

We compute a single resultant vector for red blobs, for blue blobs and for the proximity sensors:

$$\mathbf{V}_k = \sum_i \mathbf{v}_{k,i}, \qquad k = r, b, p. \tag{2}$$

Then, we rescale the vector length to be within the range $[0, 1]$ by exploiting a sigmoid normalisation:

$$\hat{\mathbf{V}}_k = \frac{\mathbf{V}_k}{|\mathbf{V}_k|} \frac{2}{1 + e^{-\beta|\mathbf{V}_k|}} - 1, \tag{3}$$

where $\beta = 2$ is a normalisation parameter. Finally, we consider the projection along $D = 6$ equally distributed axes, by computing the scalar product:

$$I_{k,d} = \hat{\mathbf{V}}_k \cdot \mathbf{V}_d, \qquad d = 1, \ldots, D, \tag{4}$$

where $\mathbf{V}_d$ is the versor in the direction $(2d - 1)\pi/D$. In this way, we reduce the total number of scalar inputs to a more manageable size without significant loss of information. The neural network has therefore 18 sensory inputs and 2 motor outputs.

The first 6 neurons receive the input from proximity sensors: $I_d = I_{p,d}$. Neurons in the range $[7, 12]$ and $[13, 18]$ receive the data corresponding to the red and blue vectors: $I_{d+6} = I_{r,d}$ and $I_{d+12} = I_{b,d}$. Finally, the output of the two motor neurons is scaled onto the range $[-\omega_m, +\omega_m]$ with $\omega_m \approx 0.34$ s^{-1}, and used to control the speed of the wheels. With 18 inputs, 2 outputs and 2 bias terms we have in total $18 \cdot 2 + 2 = 38$ genes per genotype.

Each evaluation trial is performed by initialising $N = 10$ robots randomly within a circle of 1 meter radius. All robots are provided with the same neural controller (i.e., we use a homogeneous group). The controllers are generated by the same genotype thanks to a direct encoding. Each trial lasts $T = 120$ seconds.

4.5 Strictly collaborative task

This task involves $N = 6$ robots that have to collaborate to switch off $M = 18$ beacons. Beacons are implemented as red LEDs placed over cylindrical obstacles (radius: 5 cm). Additionally, robots can emit a blue signal using their own RGB beacon. To detect beacons and signals, robots exploit their omni-directional camera. Additionally, IR proximity sensors are used for collision avoidance. Similarly to the flocking case study, the information from both camera and proximity sensors is pre-processed, resulting in $D = 6$ inputs for red blobs, blue blobs and IR proximity sensors, for a total of 18 inputs. The neural network has three outputs. The first two are scaled onto the range $[-\omega_m, +\omega_m]$ with $\omega_m \approx 0.68$ s^{-1} and used to control the wheels speed. The third output is used to control the signalling beacon, which is switched on (off) when the output is larger (smaller) than 0.5. In the whole, with 18 inputs, 3 outputs and 3 bias terms we have $18 \cdot 3 + 3 = 57$ genes per genotype.

Each evaluation trial is performed by initialising the robots randomly in a circular arena (radius: 6 m). The M beacons are also randomly positioned within the arena. The robots are homogeneous, and share identical controllers generated from a single genotype. Each trial lasts $T = 600$ seconds.

References

1. Deb K. Multi-Objective Optimization Using Evolutionary Algorithms. Chichester, UK: Wiley; 2001.

2. Coello Coello CA, Lamont GB, Van Veldhuizen DA. Evolutionary Algorithms for Solving Multi-Objective Problems. Springer, New York, NY; 2007.

3. Zitzler E, Thiele L, Laumanns M, Fonseca CM, Grunert da Fonseca V. Performance assessment of multiobjective optimizers: an analysis and review. IEEE Transactions on Evolutionary Computation. 2003;7(2):117–132.

4. Beyer HG, Schwefel HP. Evolution stratagies: a comprehensive introduction. Natural Computing. 2002;1:3–52.

5. Zitzler E, Thiele L. Multiobjective Optimization using evolutionary algorithms - A comparative case study. In: Eiben AE, Bäck T, Schoenauer M, Schwefel HP, editors. Parallel Problem Solving from Nature, PPSN V. vol. 1498 of Lecture Notes in Computer Science. Springer, Heidelberg, Germany; 1998. p. 292–301.

6. Beume N, Naujoks B, Emmerich M. SMS-EMOA: Multiobjective selection based on dominated hypervolume. European Journal of Operational Research. 2007;181(3):1653–1669.

7. Bader J, Zitzler E. HypE: An Algorithm for Fast Hypervolume-Based Many-Objective Optimization. Evolutionary Computation. 2011;19(1):45–76.

8. Igel C, Heidrich-Meisner V, Glasmachers T. Shark. Journal of Machine Learning Research. 2008 Jun;9:993–996. Available from: http://www.jmlr.org/papers/volume9/igel08a/igel08a.pdf.

9. Pinciroli C, Trianni V, O'Grady R, Pini G, Brutschy A, Brambilla M, et al. ARGoS: A Modular, Parallel, Multi-Engine Simulator for Multi-Robot Systems. Swarm Intelligence. 2012;6(4):271–295.

10. López-Ibáñez M, Paquete L, Stützle T. Exploratory Analysis of Stochastic Local Search Algorithms in Biobjective Optimization. In: Bartz-Beielstein T, Chiarandini M, Paquete L, Preuss M, editors. Experimental Methods for the Analysis of Optimization Algorithms. Berlin, Germany: Springer; 2010. p. 209–222.

11. Grunert da Fonseca V, Fonseca CM, Hall AO. Inferential Performance Assessment of Stochastic Optimisers and the Attainment Function. In: Zitzler E, Deb K, Thiele L, Coello Coello CA, Corne D, editors. Evolutionary Multi-criterion Optimization, EMO 2001. vol. 1993 of Lecture Notes in Computer Science. Springer, Heidelberg, Germany; 2001. p. 213–225.

12. Grunert da Fonseca V, Fonseca CM. The Attainment-Function Approach to Stochastic Multiobjective Optimizer Assessment and Comparison. In: Bartz-Beielstein T, Chiarandini M, Paquete L, Preuss M, editors. Experimental Methods for the Analysis of Optimization Algorithms. Berlin, Germany: Springer; 2010. p. 103–130.

13. Bonani M, Longchamp V, Magnenat S, Rétornaz P, Burnier D, Roulet G, et al. The MarXbot, a Miniature Mobile Robot Opening new Perspectives for the Collective-robotic Research. In: Proceedings of the 2010 IEEE/RSJ International Conference on Intelligent Robots and Systems (IROS 2010). Piscataway, NJ: IEEE Press; 2010. p. 4187–4193.

14. Bartz-Beielstein T, Chiarandini M, Paquete L, Preuss M, editors. Experimental Methods for the Analysis of Optimization Algorithms. Berlin, Germany: Springer; 2010.
